# Impact of brain parcellation on prediction performance in models of cognition and demographics

**DOI:** 10.1002/hbm.26592

**Published:** 2024-01-31

**Authors:** Marta Czime Litwińczuk, Nils Muhlert, Nelson Trujillo‐Barreto, Anna Woollams

**Affiliations:** ^1^ School of Health Sciences University of Manchester Manchester UK

**Keywords:** brain connectivity, cognition, demographics, parcellation, prediction

## Abstract

Brain connectivity analysis begins with the selection of a parcellation scheme that will define brain regions as nodes of a network whose connections will be studied. Brain connectivity has already been used in predictive modelling of cognition, but it remains unclear if the resolution of the parcellation used can systematically impact the predictive model performance. In this work, structural, functional and combined connectivity were each defined with five different parcellation schemes. The resolution and modality of the parcellation schemes were varied. Each connectivity defined with each parcellation was used to predict individual differences in age, education, sex, executive function, self‐regulation, language, encoding and sequence processing. It was found that low‐resolution functional parcellation consistently performed above chance at producing generalisable models of both demographics and cognition. However, no single parcellation scheme showed a superior predictive performance across all cognitive domains and demographics. In addition, although parcellation schemes impacted the graph theory measures of each connectivity type (structural, functional and combined), these differences did not account for the out‐of‐sample predictive performance of the models. Taken together, these findings demonstrate that while high‐resolution parcellations may be beneficial for modelling specific individual differences, partial voluming of signals produced by the higher resolution of the parcellation likely disrupts model generalisability.

## Graphical abstract

This study investigated how five different brain parcellation schemes impact predictive regression models and global graph theory measures. No single parcellation scheme showed a superior predictive performance of cognition and demographics. Global graph theory measures significantly differed across parcellations but did not account for differences in model predictive performance.

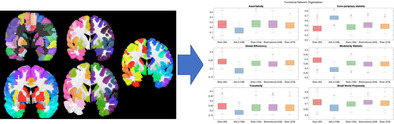

## 1 INTRODUCTION

Neuroimaging research has demonstrated that adaptive behaviour relies not only on localised activation in the brain but also on effective coordination of activity across remote neuronal populations (Alvarez & Squire, 1994; Seeley et al., 2007). To understand how information is exchanged in the human brain, research investigates the white matter connections between regions (structural connectivity [SC]) and the statistical associations between their activity (functional connectivity [FC]) (Park & Friston, 2013). SC and FC have been related to healthy cognitive function throughout the lifespan (Salami et al., 2014), whereas their disruption has been demonstrated to characterise many psychiatric, developmental and clinical diagnostics (de Kwaasteniet et al., 2013; Guye et al., 2010; Hahn et al., 2013; Keller et al., 2007; van den Heuvel & Fornito, 2014). Consequently, the study of the relationship between brain connectivity and cognition has become increasingly popular (Farahani et al., 2019; Hoppenbrouwers et al., 2014; Manca et al., 2018).

One of the most central questions in the analysis of brain connectivity is how to define the brain regions (i.e., brain parcellation) used for connectivity analysis (Eickhoff et al., 2018; Yao et al., 2015). In the analysis of brain connectivity, the brain is idealised (modelled) as a set of interconnected nodes each representing a brain region that is assumed to constitute a coherent unit of activity. The connectivity pattern between regions is then studied and often used to explain and predict cognitive function. Therefore, brain parcellation is a key step of any connectivity analysis. Nearly two dozen different brain parcellations are now available (Arslan et al., 2018; Dickie et al., 2017; Eickhoff et al., 2018; Lawrence et al., 2021). Each parcellation has been defined using different neuroimaging data and parcel generation algorithms based on different parcellation criteria. Consequently, each parcellation offers different node locations, sizes and shapes therefore leading to different representations of the activity units used for analysis. However, clinical and cognitive neuroimaging research does not yet have a standard or most common parcellation. In addition, it is unlikely that a ‘one size fits all’ standard will emerge because every parcellation method has been defined to highlight specific properties of the brain (Eickhoff et al., 2018). To illustrate, we may choose to parcellate the brain based on histological boundaries (Amunts & Zilles, 2015; Brodmann, 1909) or clustering of a group of similarly active voxels (Cohen et al., 2008). Such choices appear to impact consistency of research findings (Dhamala et al., 2021; Mellema et al., 2020; Ota et al., 2014; Pervaiz et al., 2020).

Despite the lack of an accepted standard, parcellation choice is an important decision in network analysis and it carries profound consequences for interpretation of results and comparison across different studies. It has been empirically demonstrated that parcellation can impact the properties of the estimated brain networks. For example, the parcellation used can impact the small world coefficient of SC and FC, which refers to the network's tendency to segregate into clusters of strongly connected nodes relative to its tendency to produce short paths between any pair of nodes (Fornito., 2010; Wang et al., 2009; Zalesky et al., 2010). In addition, the ability to produce highly connected nodes (aka degree distribution) can also be affected by the specific parcellation used (Fornito., 2010; Wang et al., 2009; Zalesky et al., 2010). Similarly, the choice of parcellation resolution has also been demonstrated to impact the similarities between structural and functional connectivity (Akiki & Abdallah, 2019; Ashourvan et al., 2019; Honey et al., 2009).

Parcellation choice alone has been demonstrated to impact the explanatory and predictive power of neuroimaging models of cognition in healthy and clinical populations (Dhamala et al., 2021; Mellema et al., 2020; Ota et al., 2014; Pervaiz et al., 2020). In particular, some evidence suggests that high‐resolution parcellations may benefit the predictive power of connectivity models of cognition (Mellema et al., 2020; Pervaiz et al., 2020). The difference in predictive power of various brain parcellations may be related to the properties of the networks that become more prominent with specific parcellations. For example, increased small world characteristics of FC have been related to high intelligence (Hilger et al., 2017; Langer et al., 2012). Therefore, if one brain parcellation tends to generate networks with increased specific properties, then this parcellation may influence the accuracy of model predictions. However, it is also possible that difference in predictive power of specific models of cognition is related to whether specific parcellations generate accurate connectivity profiles. For example, it is likely that a functionally‐defined parcellation is less prone to partial voluming of functional signals as compared to structurally‐defined parcellation. In contrast, a structurally‐defined parcellation that considers anatomical boundaries of regions may be better suited to define SC. In support of this proposal, Dhamala et al. (2021) have found a trend that SC defined with structural parcellation yielded more effective models of crystallised cognition than SC defined with activation‐based parcellation.

However, no work has yet been conducted to assess if the parcellation method systematically influences network architecture and the explanatory and/or predictive power of models of cognition. The present work investigated this question by defining SC and FC with a variety of parcellations: the resting state activity‐defined parcellation with either 93, 184 and 278 parcels (Shen et al., 2013), the structurally‐defined parcellation (Rolls et al., 2020) and a functional parcellation (Fan et al., 2016). Our work then produced connectivity‐based predictive models of cognition and used graph theory to assess the global differences in network properties that may explain model differences in model performance. Doing so, we tested the hypotheses that (1) SC‐based models of demographics and cognition will be more effective when connectivity is defined with a structural parcellation (Rolls et al., 2020) than with a parcellation based on a functional activation (Fan et al., 2016; Shen et al., 2013), (2) FC‐based models of demographics and cognition will be more effective when connectivity is defined with a parcellation based on brain function (Fan et al., 2016; Shen et al., 2013) than with a parcellation based on structure, (3) use of high‐resolution parcellation will improve explanatory and predictive power of models of demographics and cognition regardless of connectivity type, (4) differences in model explanatory and predictive power will systematically map with differences in network organisation, as measured by graph theory.

## 2 METHODS

### 2.1 Participants

Neuroimaging and cognitive data were obtained for 250 unrelated subjects from the 1200‐subject release of the Human Connectome Project (HCP). The subjects were blindly selected from the HCP dataset by only including those subjects who have complete behavioural data and neuroimaging data (i.e., at least one T1‐weighted image, resting‐state fMRI and diffusion MRI). For consistent treatment of behavioural and neuroimaging subjects' data selection, one subject was excluded from the neuroimaging analysis due to incomplete behavioural data. The sample consisted of 138 females and 111 males in the age range of 22 and 36 years.

### 2.2 Measures of cognition

The present work used the same measures of cognition as our previous work (Litwińczuk et al., 2022). Analysed tasks included: Picture Sequence Memory, Dimensional Change Card Sort, Flanker Inhibitory Control and Attention Task, Penn Progressive Matrices, Oral Reading Recognition, Picture Vocabulary, Pattern Comparison Processing Speed, Delay Discounting, Variable Short Penn Line Orientation Test, Short Penn Continuous Performance Test, Penn Word Memory Test and List Sorting. These assessments were obtained from the Blueprint for Neuroscience Research–funded NIH Toolbox for Assessment of Neurological and Behavioral function (http://www.nihtoolbox.org) and tasks from the Penn computerised neurocognitive battery (Gur et al., 2010). Principal component analysis (PCA) with VARIMAX rotation was then used as a feature extraction method and applied to the behavioural dataset. The extracted PCA rotated components reflected specific latent cognitive domains, interpreted as Executive Function, Self‐regulation, Language, Encoding and Sequence Processing. The present work uses the PCA scores obtained previously for each cognitive domain.

### 2.3 Minimally processed neuroimaging data

The HCP provides minimally processed neuroimaging data that was used here, the data acquisition and processing pipeline has been discussed in detail by Glasser et al. (2013). All neuroimaging data were collected with a 3T Siemens ‘Connectome Skyra’ scanner that uses the Siemens 32‐channel RF receive head coil and with an SC72 gradient insert (Ugurbil et al., 2013). Here, we utilised Version 3 of the minimal processing pipeline implemented with FSL 5.0.6 (Jenkinson et al., 2012) and FreeSurfer 5.3.0‐HCP (Dale et al., 1999).

T1 weighted MR images were acquired with a 3D MPRAGE sequence (TR = 2400 ms, TE = 2.14, TI = 1000 ms, flip angle = 8°, FOV = 224 by 224 mm, voxel size = 0.7 mm isotropic). Rs‐fMRI data was collected using the gradient‐echo EPI (TR = 720 ms, TE = 33.1 ms, flip angle = 52°, FOV = 208 by 180 mm, 70 slices, thickness = 2.0 mm, size = 2.0 mm isotropic). Scans were collected in two sessions, each lasting approximately 15 min. All four rs‐fMRI scans were used, the rs‐fMRI scans were collected both in left‐to‐right and right‐to‐left directions. In addition, in the original data, spin echo phase reversed images were acquired for registration with T1 images and the spin echo field maps were acquired for bias field correction. Diffusion‐weighted MR images were acquired with spin‐echo EPI sequence (TR = 5520 ms, TE = 89.5 ms, flip angle = 78°, refocusing flip angle = 160°, FOV = 210 by 180 mm, 111 slices, thickness = 1.25 mm, size = 1.25 mm isotropic). Each gradient consisted of 90 diffusion weighting directions plus 6 b = 0. There were 3 diffusion‐weighed shells of b = 1000, 2000 and 3000 s/mm^2^. SENSE1 multi‐channel image reconstruction was used (Sotiropoulos et al., 2013).

### 2.4 Additional processing of neuroimaging data

The Neuroimaging data were further processed following the same processing pipeline as in our previous work (Litwińczuk et al., 2022). This pipeline is summarised for completeness in the next two sub‐sections. Both SC and FC were defined with five parcellation schemes that varied in their modality and resolution. For clarity, throughout this work the parcellation name will be assisted with the number of parcels:

1. Low‐resolution resting‐state functional parcellation composed of 93 parcels (Shen et al., 2013)
2. Structural parcellation known as automated anatomical labelling (AAL3) composed of 166 parcels (Rolls et al., 2020)
3. Moderate‐resolution resting‐state functional parcellation composed of 184 parcels (Shen et al., 2013)
4. Functional parcellation known as Brainnetome composed of 246 parcels (Fan et al., 2016).
5. High‐resolution resting‐state functional parcellation composed of 278 parcels (Shen et al., 2013)

#### 2.4.1 Structural data and structural connectivity calculation

The diffusion data were further analysed using the BEDPOSTX procedure in FSL, which runs Markov Chain Monte Carlo sampling to estimate probability distributions on diffusion parameters at each voxel. This information was used in the FDT module of FSL to run ROI‐to‐ROI probabilistic tractography with ProbtrackX. Tractography was run between parcels in each of the five parcellations.

During tractography, 5000 streamlines were initiated from each voxel with step length of 0.5 mm (Behrens et al., 2003; Behrens et al., 2007; Jenkinson et al., 2012). Streamlines were constrained with curvature threshold of 0.2, maximum of 2000 steps per streamline and volume fraction threshold of subsidiary fibre orientations of 0.01. A SC matrix between regions was constructed by first counting the number of streamlines originating from a seed region i that reached a target region j ($S_{\mathrm{ij}}$). These counts are asymmetric since the count of streamlines from region i to j is not necessarily equal to the count of streamlines from region j to i ($S_{\mathrm{ij}}\neq S_{\mathrm{ji}}$), but they are highly correlated for all subjects (lowest Pearson's Correlation was 0.76, p < 0.001). Based on these counts, the weight $W_{\mathrm{ij}}$ (entries of the SC matrix) between any two pairs of regions i and j was defined as the ratio of the total streamline counts in both directions ($S_{\mathrm{ij}}+S_{\mathrm{ji}}$), to the maximum possible number of streamlines that can be shared between the two regions, which is $\left(N_{i}+N_{j}\right)*5000$ (where $N_{i}$ and $N_{j}$ are the number of seed voxels in regions i and j, respectively):

$$W_{\mathrm{ij}}=\frac{\left(S_{\mathrm{ij}}+S_{\mathrm{ji}}\right)}{\left(N_{i}+N_{j}\right)*5000}.$$

Similar to previous studies, the weight $W_{\mathrm{ij}}$ can be interpreted as capturing the connection density (number of streamlines per unit surface) between nodes i and j, which accounts for possible bias due to different sizes of the seed regions (Hagmann et al., 2008; Ingalhalikar et al., 2013). Note that the SC matrix defined based on these weights is symmetric because swapping around the regions' indices does not change the result; and it is also normalised between 0 and 1, because the maximum value of the numerator can only be reached when all streamlines originating from each of region reach the other region, so that $M_{\mathrm{ij}}=N_{i}*5000$ and $M_{\mathrm{ji}}=N_{j}*5000$, which gives $W_{\mathrm{ij}}=1$.

#### 2.4.2 Functional data and functional connectivity calculation

The minimally processed rs‐fMRI data were obtained and then further processed (Glasser et al., 2013), as recommended by Nieto‐Castanon (2020) using the CONN Toolbox (Whitfield‐Gabrieli & Nieto‐Castanon, 2012). Briefly, images were realigned, a slice‐timing corrected and outlier detection of functional images for scrubbing was performed with artefact detection tools (ART, https://www.nitrc.org/projects/artifact_detect/). Grey matter, white matter, cerebrospinal fluid and non‐brain tissues were then segmented. Images were normalised and smoothed with a 6 mm full width at half maximum Gaussian kernel. Next, the data were denoised with default Conn denoising options using the anatomical component‐based noise correction procedure (Behzadi et al., 2007). This procedure removes artefactual components from the data, including noise components from cerebral white matter and cerebrospinal areas, subject‐motion parameters (Friston et al., 1996), identified outlier scans (Powell et al., 2006) and constant and first‐order linear session effects (Whitfield‐Gabrieli & Nieto‐Castanon, 2012). Then standard denoising steps were applied including scrubbing, motion regression and application of a high pass filter (0.01 Hz cut‐off), and a low pass filter (0.10 Hz cut‐off ).

FC analysis was performed based on the same five parcellations. The average blood oxygenation level‐dependent signal in each parcel was obtained and the pairwise (parcel‐to‐parcel) correlation of the averaged signals was calculated. Since the CONN toolbox produces Fisher's *Z*‐scores (Fisher, 1915), a hyperbolic tangent function was used to reverse Fisher's transformation and obtain original correlation values ranging between −1 and 1.

### 2.5 Model construction

Predictive models of five cognitive domains and three demographic variables were separately constructed using FC, SC or concatenated SC and FC matrices (referred to as combined connectivity [CC]) as predictors. Models also differed in the brain parcellation scheme used to define the network nodes for connectivity calculations. This led to a total of 120 models to be analysed ([5 cognitive domains +3 demographic characteristics]*3 connectivity types*5 parcellation schemes). Prior to model estimation, for each cognitive domain, the confounding effect of age, gender and education was regressed out of the response variable. Meanwhile, for each demographic, the remaining demographics were included in the main model as covariates of no interest. Then, all models were estimated using a principal component regression (PCR) approach with elastic net regularisation of the regression coefficients in latent space (EN‐PCR). The overall estimation procedure consisted of the following steps:

1. PCA decomposition was used to orthogonalise the predictors' (connectivity) matrix
2. A regression model in latent (PCA) space was fitted with elastic net regularisation for the regression coefficients
3. Regression coefficients obtained in PCA space were projected back to the original connectome space and used to produce predicted responses.

To tune the elastic net regularisation hyper‐parameters alpha and lambda and evaluate the out‐of‐sample model performance, a Bootstrap bias corrected cross‐validation (BBC‐CV) approach was used (Tsamardinos et al., 2018). In brief, the BBC‐CV consisted in a repeated (50 times) 5‐fold cross‐validation with hyperparameter tuning (CVT), followed by a Bootstrap bias correction procedure (5000 bootstrap samples). The later Bootstrap step accounts for optimistic biases in the estimation of model performance introduced by using the same data for both hyperparameter tuning and model evaluation in the CVT step (Tsamardinos et al., 2018). The explained variation (coefficient of determination) between the predicted and observed responses was used as the statistic for both hyperparameter tuning and out‐of‐sample performance evaluation of the model (Poldrack et al., 2020). The resultant population of bootstrapped statistics was used to produce mean performance estimates of the EN‐PCR learning strategy and corresponding confidence intervals. For a similar application of the BBC‐CV method in the context of predictive modelling of cognition based on connectivity we refer the authors to our previous paper (Litwińczuk et al., 2022).

Finally, a permutation test was used to assess how likely it is to get the observed models' performance by chance. Specifically, the out‐of‐sample predictions produced by the CVT (249 participants) were sampled without replacement 5000 times. Each time, the entries in the observed response variable were permuted. Then, the models' coefficient of determination was estimated. This permuted distribution was compared with the distribution of coefficients of determination obtained for the non‐permuted data. That is, the *p*‐value for testing the significance of models' performance was determined by computing the proportion of permuted statistics at least as high as or greater than the observed statistics.

### 2.6 Model comparison

All following analysis was done separately for each cognitive domain and connectivity type, and the analysis compared the effects of parcellation choice.

To compare the models' whole‐sample explanatory power, the Akaike information criterion (AIC) for each model was obtained. The AIC balances the goodness‐of‐fit of the model (model accuracy) against its complexity (the number of estimable parameters of the model) so that a reduced AIC is associated with improved model quality. Given any two models *M*_1_ and *M*_2_, a positive difference (ΔAIC = AIC [*M*_1_]−AIC [*M*_2_]) is interpreted as weak (1–2 units), strong (2–4 units), considerably strong (4–7 units) and decisive (>10) evidence against evidence against *M*_1_ (Burnham & Anderson, 2016). We used the AIC difference to compare the explanatory power of alternative models of the same response variable (cognitive or demographic) with the same connectivity modality as predictor (SC, FC or CC). The models under comparison only differed in the type of parcellation used to compute the connectivity predictors. Consequently, if two parcellations had similar explanatory power but one was coarser than the other, the coarser parcellation was favoured due to requiring fewer parameters (number of connectivity predictors) to model the same response data.

To compare the models' out‐of‐sample predictive performance, the models' out‐of‐sample predictions (generated during repeated BBC‐CVT) were used to obtain a bootstrapped (5000 samples) estimates of the models' performance statistics (coefficient of determination). Additionally, the non‐parametric Wilcoxon rank sum test for equal medians was used to assess the significance of differences in performance between different models. These comparisons were only done for models which performed better than chance.

### 2.7 Graph theory measures

Graph theoretic measures were calculated based on the weighted, undirected SC and FC matrices of every subject, using The Brain Connectivity Toolbox (http://www.brain-connectivity-toolbox.net). Measures of global network organisation included small‐world propensity, global efficiency, assortativity, modularity statistic, transitivity and coreness statistic resulting from core‐periphery partition. Small world propensity requires computation of the clustering coefficient and the shortest path length of the network (Muldoon et al., 2016). The clustering coefficient was obtained using Onnela's algorithm (Muldoon et al., 2016; Onnela et al., 2005) whereas the shortest path length was obtained using the Floyd–Warshall Algorithm applied to the weighted graph obtained from the inverse of each connectivity matrix. Modularity statistic requires computation of network modules, which were defined with Newman's algorithm (Newman, 2006).

To study the impact of the parcellation on global network organisation, a series of paired t‐tests were used to test for significant differences between the same global graph theoretic measure computed for two different parcellation schemes. To explore the impact of global network architecture yielded by each parcellation on predictive modelling, regression models were fitted to each observed cognitive domain using graph theory. Graph theory models were fitted and compared in the same manner as for connectivity but without elastic‐net regularisation.

## 3 RESULTS

### 3.1 Connectivity

#### 3.1.1 Predictive modelling of demographics

**FIGURE 1:**
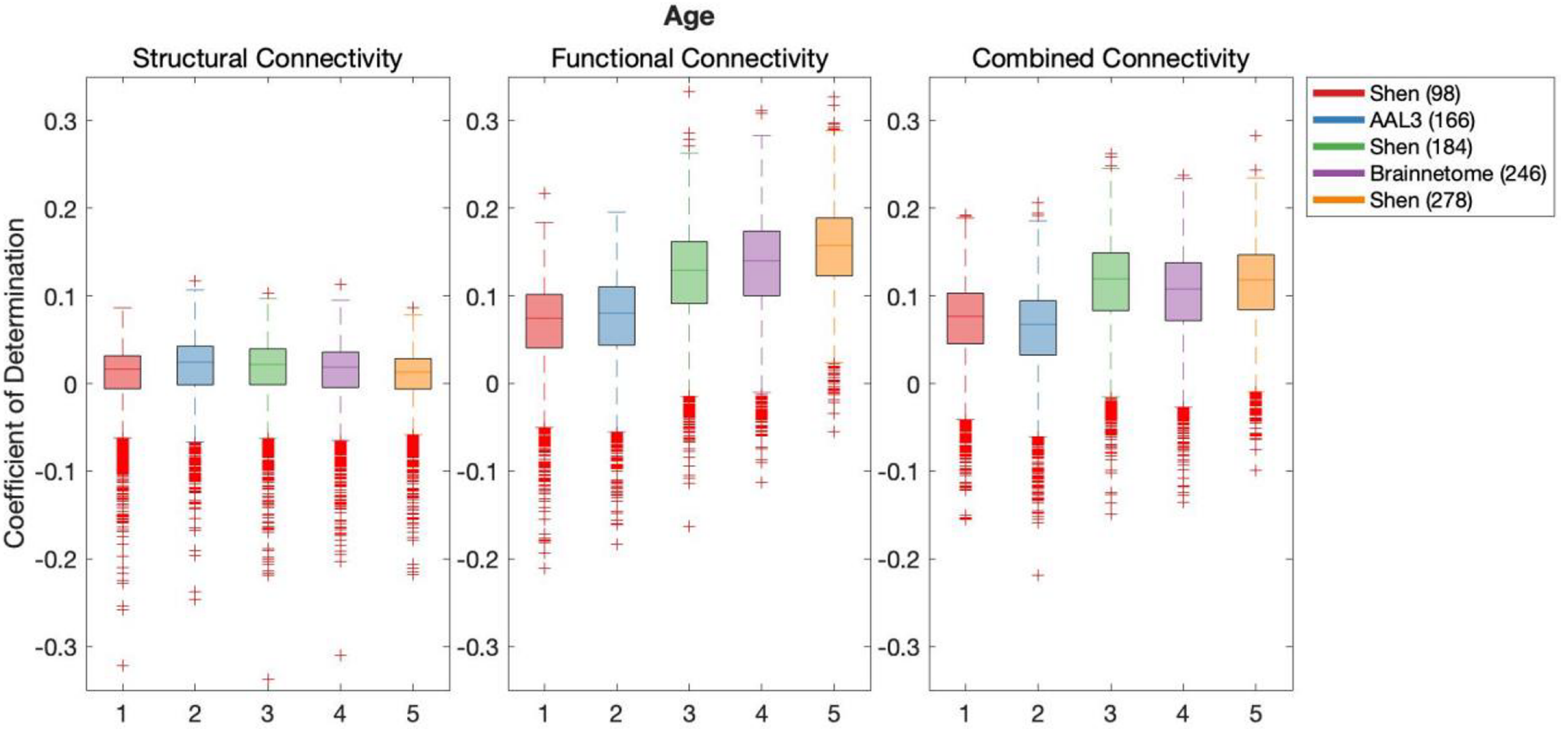
Results of bootstrap bias corrected cross‐validation of age models constructed with structural connectivity, functional connectivity and combined connectivity, as measured by the coefficient of determination. The solid lines show the median scores, the boxes show the interquartile range and ticks outside of the whiskers indicate outlier scores across all bootstrap samples. Filled boxes illustrate above‐chance predictive performance and unfilled boxes illustrate below‐chance prediction.

**FIGURE 2:**
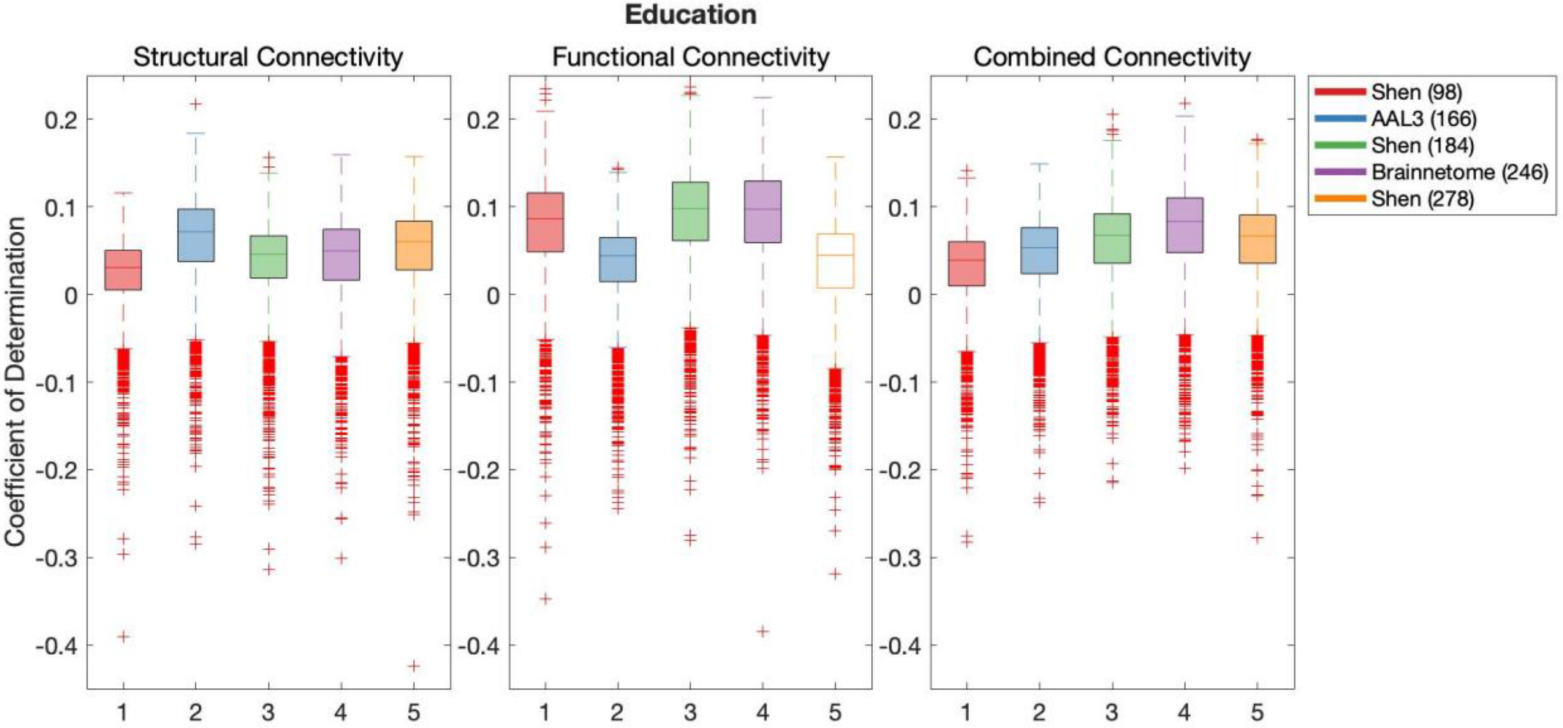
Results of bootstrap bias corrected cross‐validation of education models constructed with structural connectivity, functional connectivity and combined connectivity, as measured by the coefficient of determination. The solid lines show the median scores, the boxes show the interquartile range and ticks outside of whiskers indicate outlier scores across all bootstrap samples. Filled boxes illustrate above‐chance predictive performance and unfilled boxes illustrate below‐chance prediction.

**FIGURE 3:**
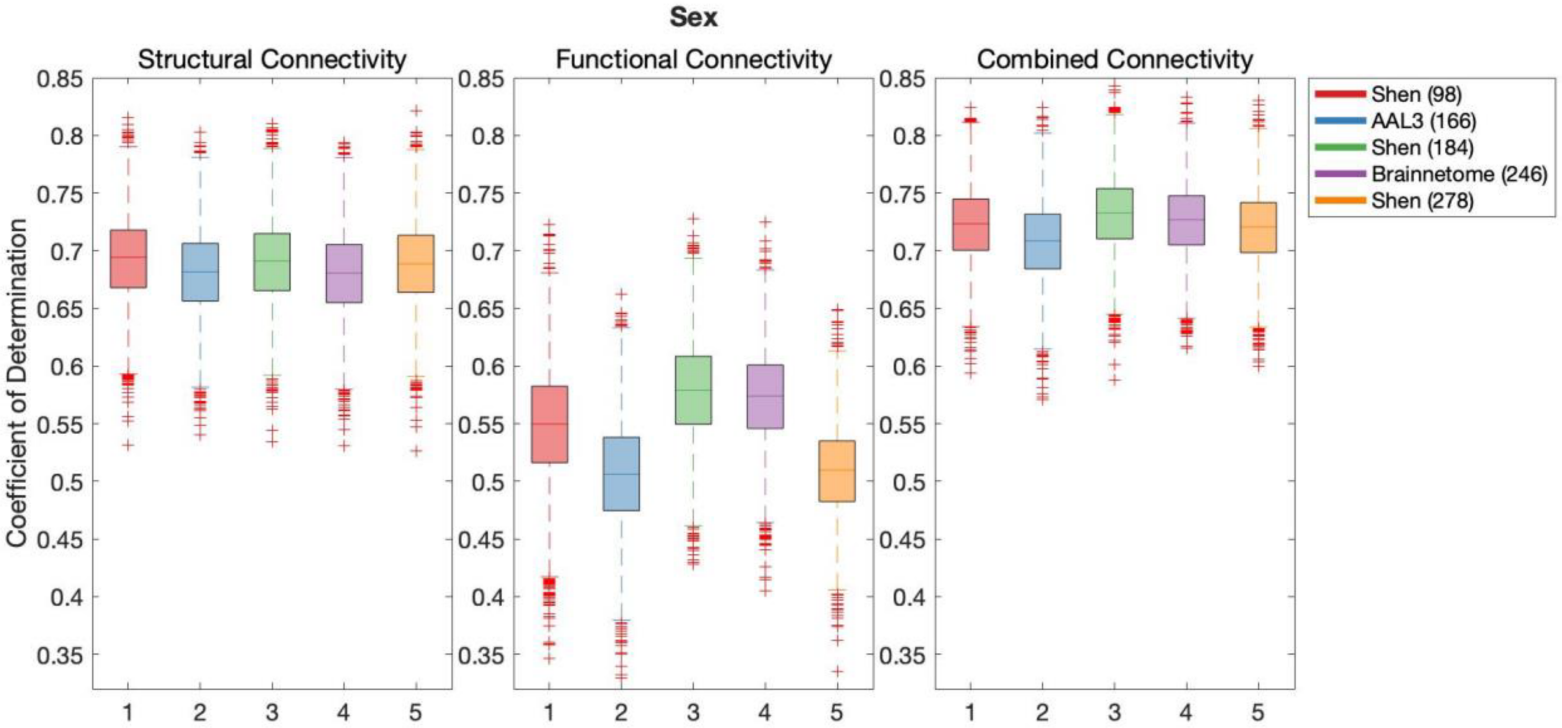
Results of bootstrap bias corrected cross‐validation of sex models constructed with structural connectivity, functional connectivity and combined connectivity, as measured by the coefficient of determination. The solid lines show the median scores, the boxes show the interquartile range and ticks outside of whiskers indicate outlier scores across all bootstrap samples. Filled boxes illustrate above‐chance predictive performance and unfilled boxes illustrate below‐chance prediction.

Results of the cross‐validation of models of demographics are presented in Figures 1, 2, 3. Cross‐validation results demonstrated that only FC defined using Shen (278) parcellation did not produce above chance out‐of‐sample predictions of Education. All remaining models of all demographic variables performed above chance regardless of the parcellation schemes or the connectivity modality used as predictor.

Results of Wilcoxon rank sum test comparing out‐of‐sample models' performance are shown in Table S1, where the false discovery rate (FDR) adjusted significance threshold equals 0.0095 (Benjamini & Hochberg, 1995). The following results and effect sizes (Rosenthal et al., 1994) have been written for significant pair‐wise comparisons presented in Table S1.

Out‐of‐sample predictive performance of age was significantly higher for models using AAL3 (166) parcellation in SC than any other parcellation, for Shen (278) in FC and for Shen (184) in CC. In the case of education, out‐of‐sample predictive performance was also significantly higher for models using AAL3 (166) parcellation in SC than alternative parcellations. In FC, highest out‐of‐sample predictive performance for education was found for both Shen (184) and Brainnetome (246), and there was no significant difference between their prediction performance. For CC, Brainnetome (246) parcellation performed more effectively at predicting education than alternative parcellations. Finally, Sex was most effectively predicted using Shen (93) parcellation in SC, and Shen (184) in both FC and CC.

#### 3.1.2 Predictive modelling of cognition

**FIGURE 4:**
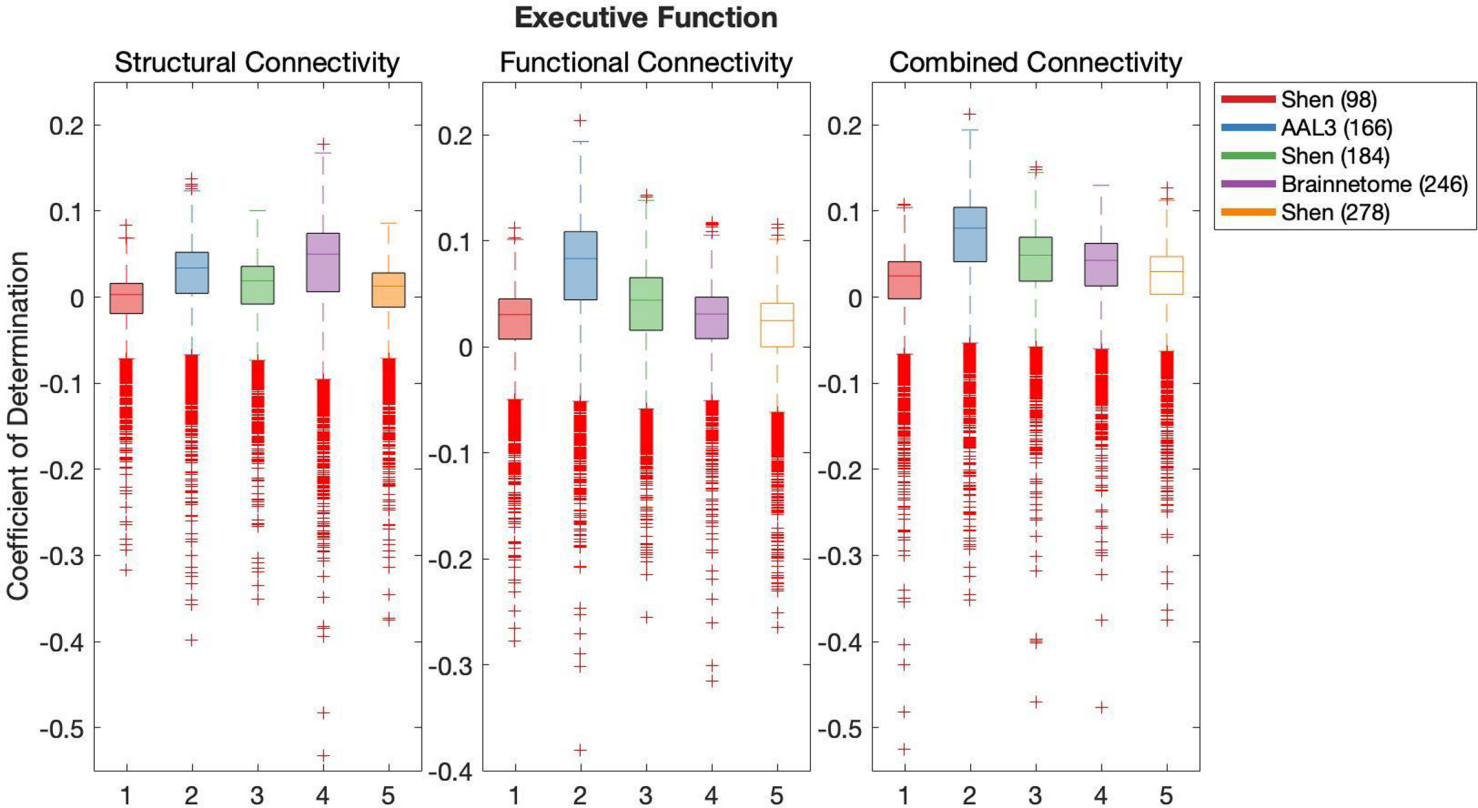
Results of bootstrap bias corrected cross‐validation of executive function models constructed with structural connectivity, functional connectivity and combined connectivity, as measured by the coefficient of determination. The solid lines show the median scores, the boxes show the interquartile range and ticks outside of whiskers indicate outlier scores across all bootstrap samples. Filled boxes illustrate above‐chance predictive performance and unfilled boxes illustrate below‐chance prediction.

**FIGURE 5:**
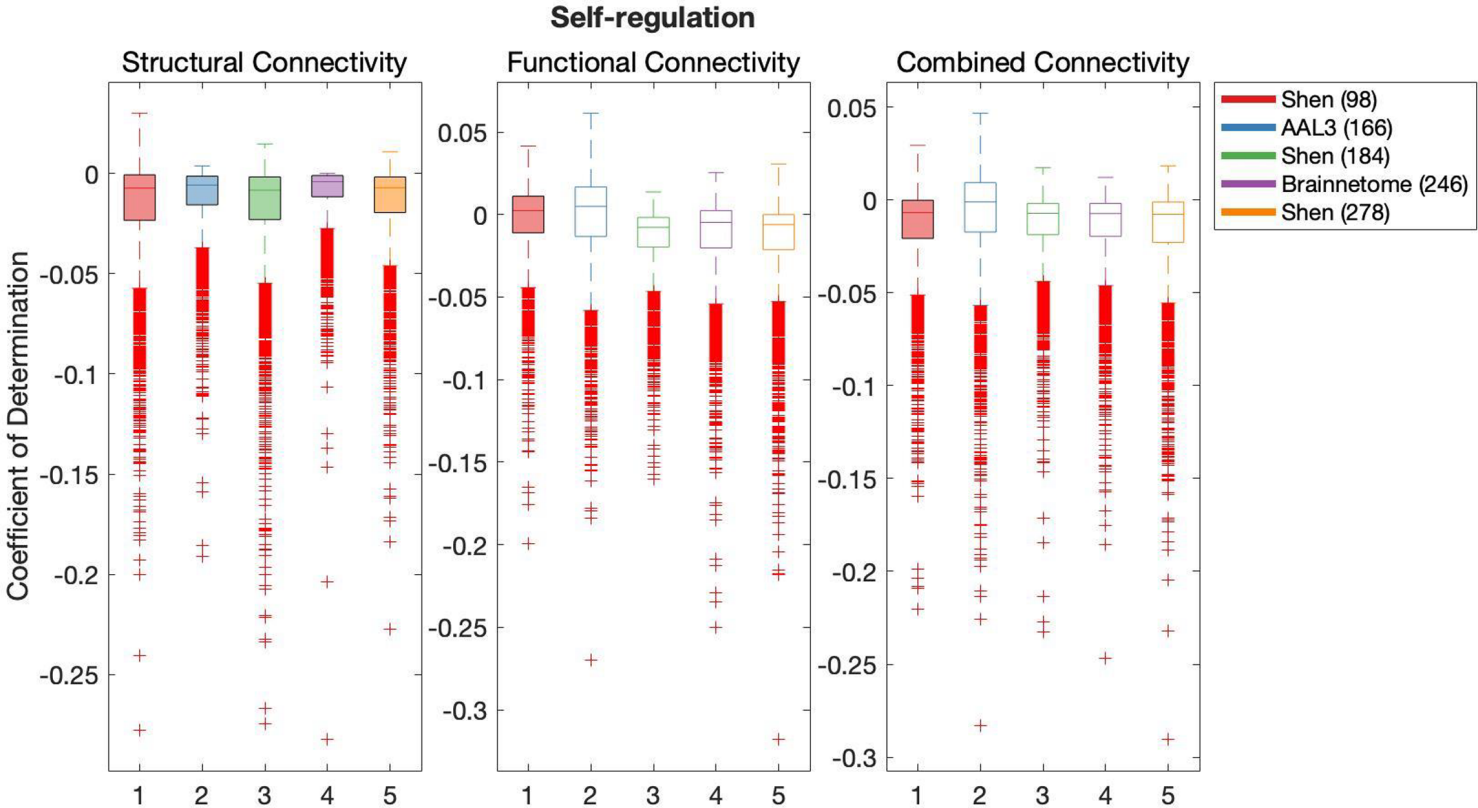
Results of bootstrap bias corrected cross‐validation of self‐regulation models constructed with structural connectivity, functional connectivity and combined connectivity, as measured by the coefficient of determination. The solid lines show the median scores, the boxes show the interquartile range and ticks outside of whiskers indicate outlier scores across all bootstrap samples. Filled boxes illustrate above‐chance predictive performance and unfilled boxes illustrate below‐chance prediction.

**FIGURE 6:**
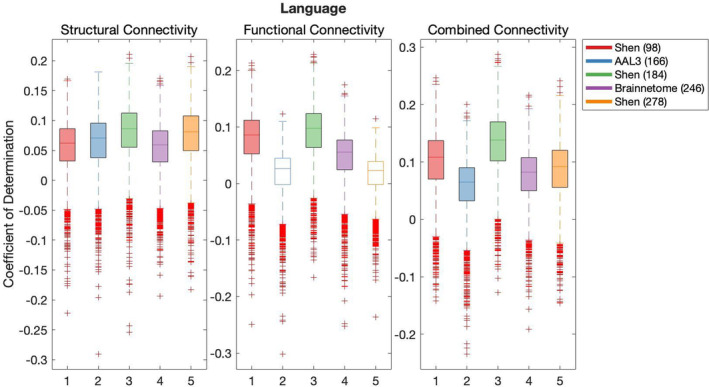
Results of bootstrap bias corrected cross‐validation of language models constructed with structural connectivity, functional connectivity and combined connectivity, as measured by the coefficient of determination. The solid lines show the median scores, the boxes show the interquartile range and ticks outside of whiskers indicate outlier scores across all bootstrap samples. Filled boxes illustrate above‐chance predictive performance and unfilled boxes illustrate below‐chance prediction.

**FIGURE 7:**
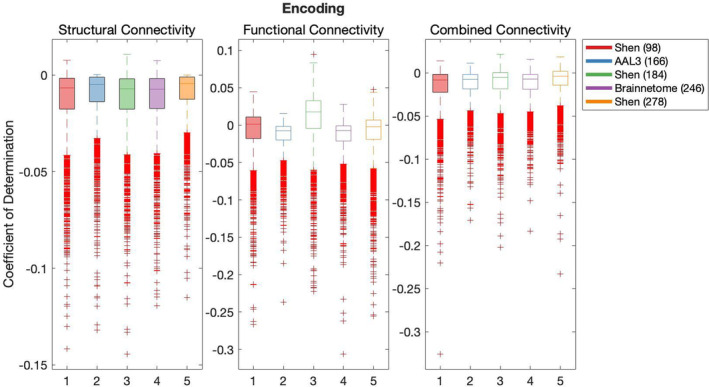
Results of bootstrap bias corrected cross‐validation of encoding models constructed with structural connectivity, functional connectivity and combined connectivity, as measured by the coefficient of determination. The solid lines show the median scores, the boxes show the interquartile range and ticks outside of whiskers indicate outlier scores across all bootstrap samples. Filled boxes illustrate above‐ chance predictive performance and unfilled boxes illustrate below‐chance prediction.

**FIGURE 8:**
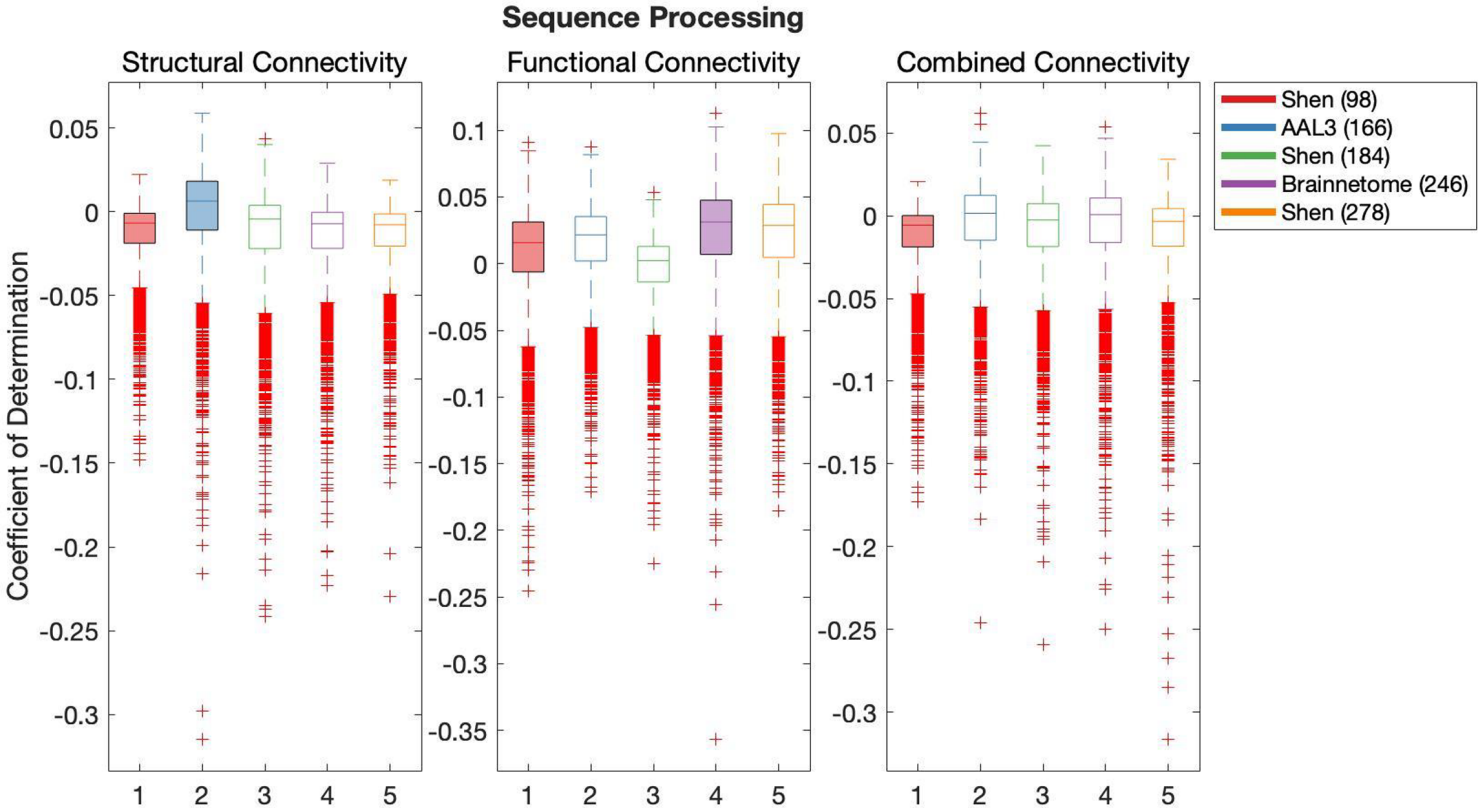
Results of bootstrap bias corrected cross‐validation of sequence processing models constructed with structural connectivity, functional connectivity and combined connectivity, as measured by the coefficient of determination. The solid lines show the median scores, the boxes show the interquartile range and ticks outside of whiskers indicate outlier scores across all bootstrap samples. Filled boxes illustrate above‐chance predictive performance and unfilled boxes illustrate below‐chance prediction.

Results of the cross‐validation of models of cognition are presented in Figures 4, 5, 6, 7, 8. It was found that the Shen (93) parcellation consistently produced out‐of‐sample predictions of cognition that explained more variance in unseen samples than chance. This was observed across SC, FC and CC.

All pairwise comparisons of the cross‐validation performance are presented in Table S2. The probability of pairwise comparisons was adjusted with FDR (Benjamini & Hochberg, 1995). All following comparisons have passed the significance threshold of 0.0184.

Despite consistently explaining more variance in unseen sample than chance, Shen (93) rarely outperformed other parcellations at producing effective predictions of cognition (vs. Shen 184 Self‐regulation structural connectivity, Language versus Brainnetome (246) structural connectivity, functional connectivity and combined connectivity, Language versus AAL3 (166) with CC and Language versus Shen 278 with CC). Next, AAL3 (166) consistently produced the most effective predictions of Executive Function utilising FC and CC. Similarly, Brainnetome (246) consistently produced the most effective predictions of Self‐regulation utilising SC. Shen (184) parcellation consistently produced the most effective predictions of Language utilising FC. Shen (278) parcellation consistently produced the most effective predictions of Encoding utilising SC. There was no other parcellation scheme that consistently outperformed other parcellation schemes for any given type of connectivity or any cognitive construct.

### 3.2 Graph theory

#### 3.2.1 Comparison of global organisation across parcellations

Figures S17 and S18 show the distribution of scores obtained for each participant's global graph theory measures for each parcellation. Tables S3 and S4 present pair‐wise comparisons made to assess differences in global graph theory measures across parcellations. It was generally found that parcellation schemes significantly impacted network organisation, as measured by all six graph theory measures. However, the direction of this impact was not necessarily shared across SC and FC. For example, structural parcellation scheme (AAL3, 166) produced high modularity statistic in SC, but low modularity statistic in FC. Further, high resolution scheme (Shen, 278) produced lowest global efficiency and transitivity in SC, whereas in FC a lower resolution parcellation (AAL3, 166) produced lowest global efficiency and transitivity.

#### 3.2.2 Predictive modelling of demographics and cognition

Results of the cross‐validation of models of demographics are presented in Figures S19–S26. It was found that graph theory measures of global organisation of SC, FC and CC could not produce out‐of‐sample predictions of demographics or cognition that would explain more variance in unseen samples than chance.

## 4 DISCUSSION

Currently available brain parcellation schemes vary in the neuroimaging data and the node‐generation algorithms used to obtain them. Consequently, the obtained parcellations vary in the number, size and shape of the nodes. These differences have been demonstrated to largely impact the predictive power of connectivity‐based models of cognition (Dhamala et al., 2021; Mellema et al., 2020; Ota et al., 2014; Pervaiz et al., 2020). However, to date, it remained unclear if the method of parcellation is systematically impacting predictive models. To fill this gap in the literature, the present research defined SC, FC and CC with five different popular parcellation schemes and then constructed predictive models of demographics and cognition based on models using each connectivity modality and parcellation scheme as predictors. Results of AIC‐based model comparison did not show a notable benefit to choosing a specific parcellation to model sample demographics. However, there was some trend towards the benefit of using the low‐resolution functional parcellation (Shen 93) to model cognition. Analysis of the out‐of‐sample performance of the models showed that most parcellations produced above‐chance predictive performance of demographics for all connectivity modalities. The only exception to this was the model of educational level using FC with the Shen 278 parcellation. In addition, it was found that models of demographics showed higher performance when using FC defined with functional parcellations. There was no consistent benefit to using low or high‐resolution parcellations for this connectivity modality. When SC was used to model age and education, AAL3 (166) parcellation produced the highest out‐of‐sample performance. In contrast, models of sex based on SC modality benefitted from using low‐resolution functional parcellation (Shen, 93). When out‐of‐sample predictions of cognition were assessed, it was found that no parcellation scheme would consistently produce significantly higher predictive performance than the others, even when the connectivity and parcellation corresponded to the same modality (functional or structural). However, out‐of‐sample predictions of cognition were consistently above chance when using connectivity defined with the Shen 93 parcellation scheme. This was the only parcellation scheme that achieved such consistency in performance.

Prior research has demonstrated that effective modelling of cognition to some extent depends on parcellation. For example, Dhamala et al. (2021) reported that SC defined with low‐resolution FreeSurfer parcellation succeeded in producing predictions of crystallised intelligence in an unseen sample. However, the same connectivity failed to produce effective predictions when in‐house high‐resolution parcellation (CoCo 439) was used instead. In contrast, FC tended to benefit at predicting cognitive abilities when CoCo 439 parcellation was used. Their CoCo 439 parcellation was defined from functional data suggesting a benefit of using a parcellation that corresponds to the modality of connectivity. However, in the present report, when individual cognitive domains were analysed, such benefits were lost. For example, we found that Executive Function was most effectively modelled with FC (as reflected by out‐of‐sample predictions), when FC was defined with AAL3 (166) parcellation—a parcellation defined with neural anatomy. In addition, similarly to the report from Dhamala et al. (2021), this work could not identify a single superior parcellation. Such inconsistency of findings warrants caution when interpreting results obtained with predictive modelling. Authors must consider that any superiority of a given model defined with a given set of data may be related to the parcellation scheme used. It does remain possible that some alternative publicly available parcellation scheme exists that would emerge as superior to others, but so far there is no evidence to suspect that such parcellation exists.

In another seminal report, Pervaiz et al. (2020) demonstrated that the most accurate predictions of fluid cognition were produced with models defined with high‐resolution parcels generated by spatial independent component analysis. With this algorithm, parcels can overlap and are not necessarily contiguous. This contrasts with publicly available parcellations used in the present work, which were defined to identify non‐overlapping, contiguous parcels. Consequently, the parcellations offered with fewer constraints may prove to produce more accurate parcels with coherent signals (i.e., avoid partial voluming of signals). For example, recent work has demonstrated graded changes in connectivity patterns across cortex (Bajada et al., 2017; Cloutman et al., 2020), which suggests that smooth parcel boundaries may better describe connectivity of the system. This need for overlapping parcels may explain why the results presented by Pervaiz et al. (2020) identified high resolution to benefit modelling of fluid cognition, while our results could not identify this pattern. However, Pervaiz et al. (2020) have not assessed the predictive value of various parcellation schemes in modelling done with different parcellations with SC or CC. The present work adds to the field the finding that SC and CC do not benefit in predictive power from modality‐corresponding parcellation or a specific resolution of parcellation.

A key consideration in the future may be the choice of parcellation scheme. In the present work, we found that demographics and cognition were well predicted by a low‐resolution functional parcellation scheme (Shen, 93). This was observed regardless of whether SC, FC or CC was used to predict demographics and cognition. One explanation of this finding is that low resolution parcellations may be less prone to partial voluming of signals, due to individual differences in neural anatomy and function. However, we also found that some high‐resolution parcellation schemes, when accurate, always produced more generalisable models. This highlights that low‐resolution parcellations may offer some insights into neural substrates of cognition and high‐resolution parcellations further complement this information. In addition, specific parcellation schemes had been shown to lead to differences in identifying functionally homogeneous regions which could impact on accuracy of predictive modelling (Peng et al., 2023; Schaefer et al., 2018). Future work can systematically explore exactly how different parcellation schemes impact prediction accuracy.

A key finding of this work was that although parcellation schemes impacted the global architecture of brain networks, the global architecture of the networks had no out‐of‐sample predictive power with demographics or cognition. This suggests that global brain organisation measures reflect changes in the organisation across parcellation schemes but this change alone cannot account for the reason why specific parcellation schemes excel at modelling demographics or cognition. At the moment, partial voluming of signals presents a much stronger explanation of why specific parcellations succeed at modelling cognition and others fail. In the future, it will be possible to implement in‐house parcellations that allow for overlapping parcels and manipulate the resolution of parcellation (e.g., spatial Independent Component Analysis, spectral clustering and spectral‐reordering algorithm), to generate different parcellations and investigate further if organisational properties revealed by specific resolution of parcellation relate to demographics and cognition.

One key limitation of the present work is that we have not considered alternative resolutions of parcellations defined with anatomical or a hybrid of anatomical and functional information. In particular, we have investigated four resolutions of functional parcellation but only a single resolution of anatomical parcellation. It is possible that investigations of alternative resolutions in these corresponding modalities of connectivity would reveal a distinct benefit for predictive modelling with SC or CC. For example, it is possible that high‐ or low‐resolution SC is particularly important for the prediction of cognitive domains, and we failed to capture these benefits because we only varied resolution for the functional parcellation scheme. A functional parcellation scheme is likely poorly suited to accurately identify parcel volumes in structural connectivity and it may have missed the benefit of various resolutions of structural parcellations. Another key caveat is that in principle some of the quantitative results might differ by employing different regression methods. However, it is expected that the main message of this work (i.e., that parcellation schemes do not introduce a systematic bias to modelling) would remain valid. This is evidenced by the fact that the accuracy results presented here are comparable to the ones obtained using other linear regression methods (Dhamala et al., 2021; Rasero et al., 2021).

Overall, this paper has investigated whether the method of parcellation is systematically related to the efficiency of predictive models of cognition and parcellation. By investigating the generalisability of SC, FC and CC predictive models defined with five different parcellations, we have found that low‐resolution parcellation (Shen 93) was consistently able to produce generalisable models of demographics and cognition. However, when alternative parcellations succeeded at producing generalisable models then these parcellations outperformed the Shen 93 parcellation. It remained challenging to identify a superior parcellation scheme. As a result of this work, it is important that from here onwards authors provide a cautious interpretation of superior models of cognition based on connectivity, as specific parcellation schemes may unsystematically impact the results.

## FUNDING INFORMATION

Biotechnology and Biological Sciences Research Council, UK (grant reference: BB/M011208/1). Data were provided by the Human Connectome Project, WU‐Minn Consortium (Principal Investigators: David Van Essen and Kamil Ugurbil; 1U54MH091657) funded by the 16 NIH Institutes and Centers that support the NIH Blueprint for Neuroscience Research; and by the McDonnell Center for Systems Neuroscience at Washington University.

## CONFLICT OF INTEREST STATEMENT

None of the authors have a conflict of interest to disclose.

## Supporting information


**DATA S1:** Supporting Information.

## Data Availability

Data are openly available as part of the WU‐Minn HCP 1200 Subjects Data Release of HCP Young Adult study, part of the Human Connectome Project (https://www.humanconnectome.org/study/hcp-young-adult/). Codes for data analysis are available at https://github.com/MCLit/atlas.
