# Supplementary material for: Impact of brain parcellation on prediction performance in models of cognition and demographics
Source: Hum Brain Mapp. 2024 Jan 31;45(2):e26592. doi: 10.1002/hbm.26592 (PMC10831203; doi:10.1002/hbm.26592)
Supplement: Supplementary file 1 — DATA S1: Supporting Information. [file HBM-45-e26592-s001.docx]

## Connectivity

### Predictive modelling of demographics

#### Relative predictive performance

Table 1 Pairwise comparison of Z-scores of coefficients of determination of demographics generated during BBC-CVT. Parcellations presented in rows were used as the reference, therefore positive values reflect that the parcellation presented in a given row performs better than the parcellation presented in a given column, whereas negative values indicate that the parcellation presented in a given row performs poorer than the parcellation presented in a given column. Comparisons were only performed for parcellations that produce better predictions than chance. All Z-values that are presented in bold meet the FDR-corrected p-value of 0.0095.

#### AIC-based model comparison

AIC values demonstrated that across all connectivity modalities and parcellations, age was best modelled with SC defined either with AAL3 (166) or Shen (93) parcellations (Figure 1). The difference between AIC values of SC models defined with either AAL3 (166) or Shen (93) was small. For SC, there was evidence in favour of both AAL3 (166) and Shen (93) parcellations relative to Shen (184) and Shen (278), and Brainnetome (246). For FC, there was substantial evidence in favour of Shen (93) parcellation to AAL3 (166). For CC, there was substantial evidence in favour of Shen (93) parcellation above to Shen (184).

Next, across connectivity modalities and parcellations, education had lowest AIC value when modelled with SC defined with Shen (93) (Figure 2). Within SC, there was evidence in favour of Shen (93) relative to AAL3 (166), Shen (278) and Brainnetome (246) and substantial evidence in favour of Shen (184). For FC, Brainnetome (246) was favoured to Shen (93). For CC, Shen (93) was favoured above Shen (184) and Shen (278).

Finally, there was no evidence to suggest that sex could be modelled more effectively using any single connectivity modality or parcellation (Figure 3).


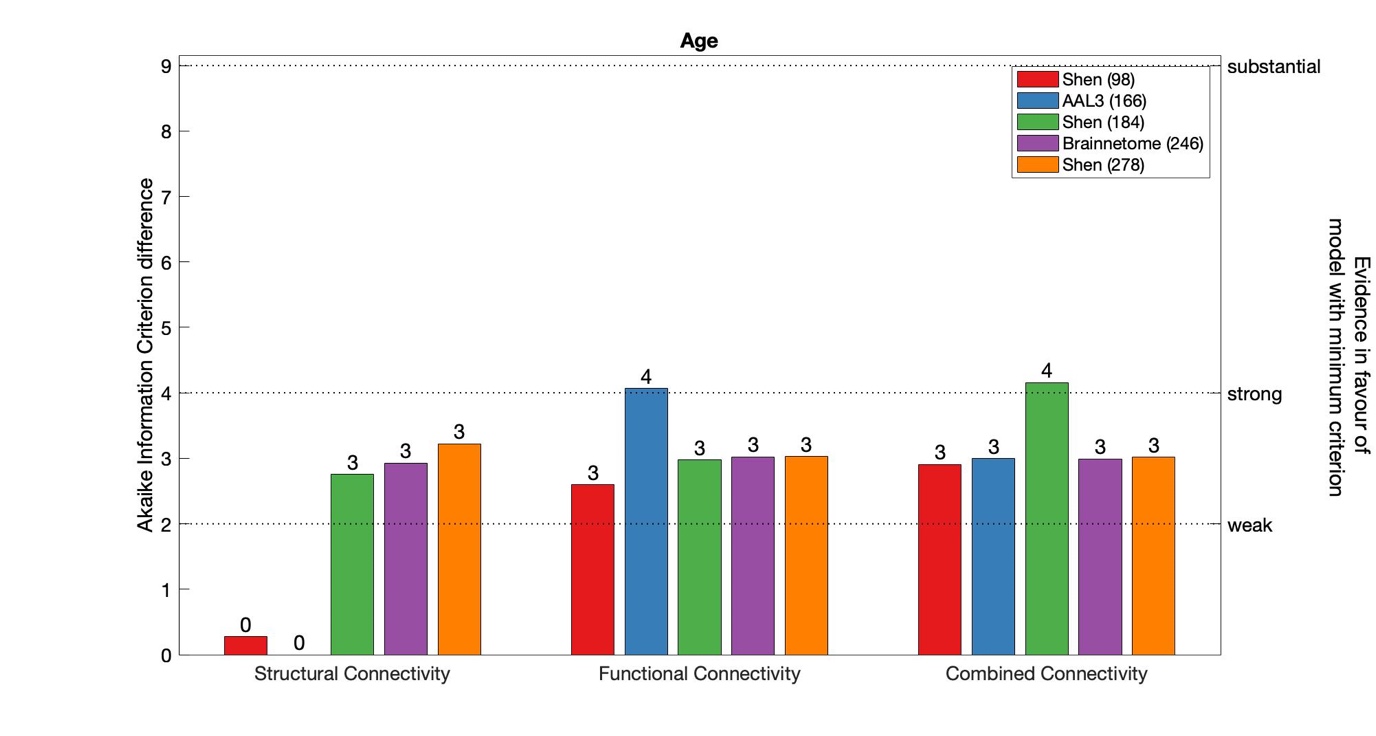


Figure 1 AIC difference for SC, FC and CC models of age, constructed with each parcellation scheme. Dotted line marks substantial evidence in favour of SC model defined with Shen (246) (minimum AIC across all modalities and parcellations).


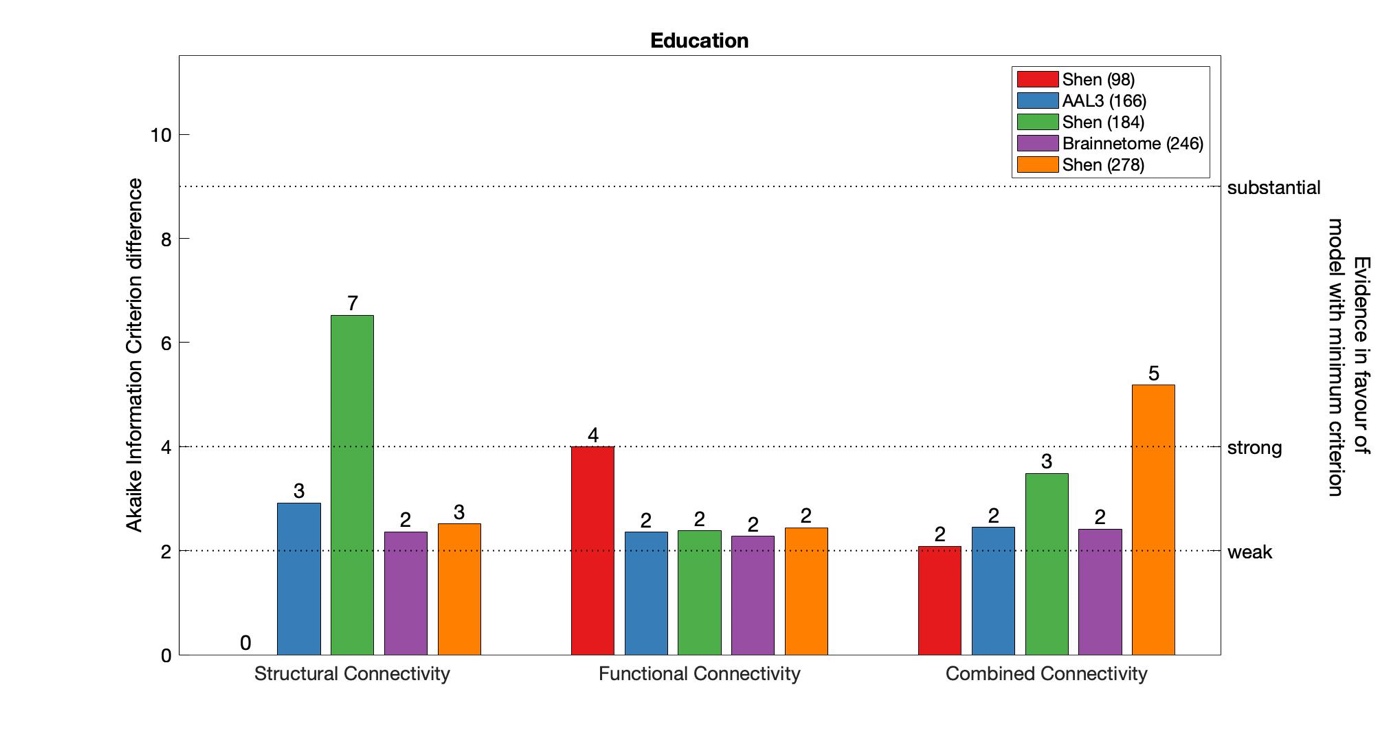


Figure 2 AIC difference for SC, FC and CC models of education, constructed with each parcellation scheme. Dotted line marks substantial evidence in favour of CC model defined with Shen (246) (minimum AIC across all modalities and parcellations).


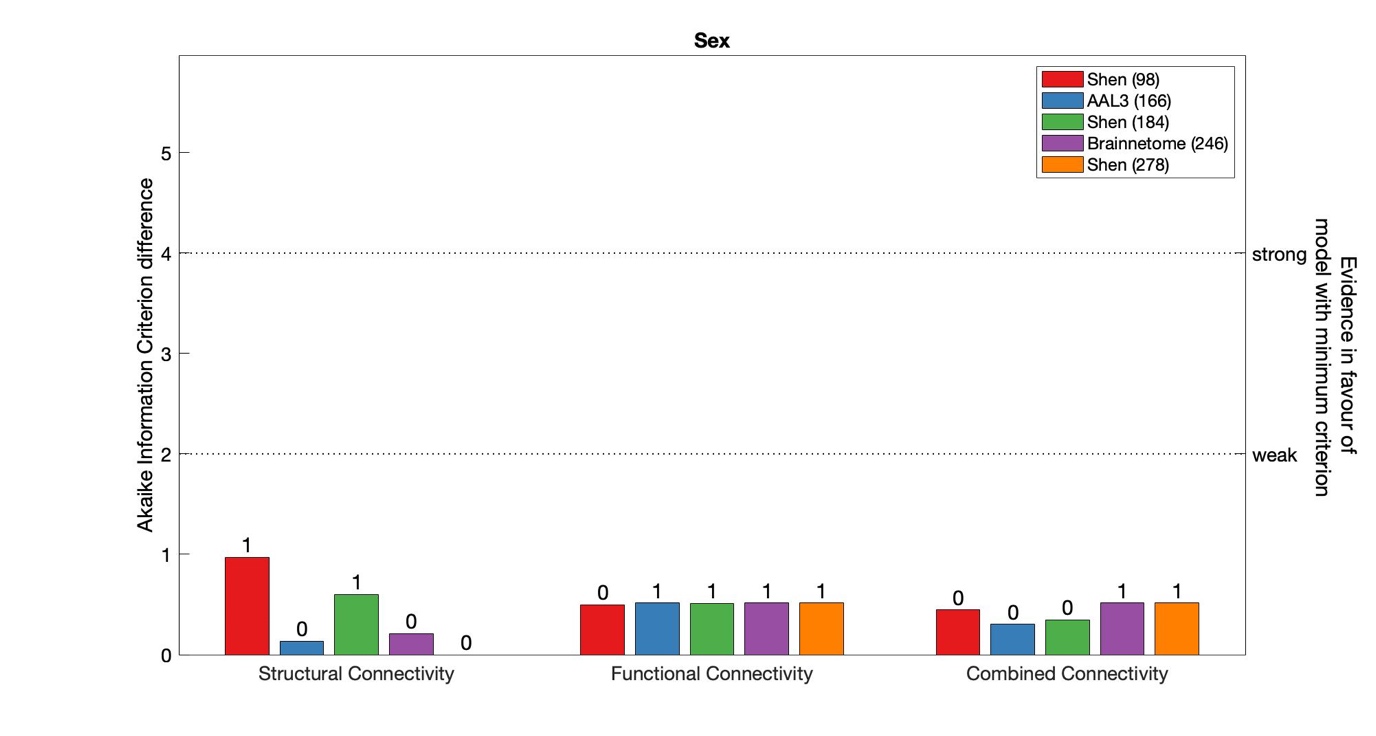


Figure 3 AIC difference for SC, FC and CC models of sex, constructed with each parcellation scheme. Dotted line marks substantial evidence in favour of CC model defined with Shen (246) (minimum AIC across all modalities and parcellations).

### Predictive modelling of cognition

Table 2 Pairwise comparison of Z-scores of coefficients of determination of cognition generated during BBC-CVT. Parcellations presented in rows were used as the reference, therefore positive values reflect that the parcellation presented in a given row performs better than the parcellation presented in a given column, whereas negative values indicate that the parcellation presented in a given row performs poorer than the parcellation presented in a given column. Comparisons were only performed for parcellations that produce better predictions than chance. All Z-values that are presented in bold have the p-value <0.0184.

#### AIC-based model comparison

Figures 4-8 demonstrate AIC for models of cognition composed with SC, FC and CC defined with the 5 parcellation schemes.

AIC favoured models of Executive Function composed with FC defined with AAL3 (166) parcellation. For SC, there was substantial evidence in favour of Brainnetome (246) parcellation. For both FC and CC, there was substantial evidence in favour of AAL3 (166) parcellation.

There was substantial evidence in favour of FC model of Self-regulation constructed with AAL3 (166). In addition, for SC, there was substantial evidence in favour of Shen (184) parcellation. For CC, there was substantial evidence in favour of AAL3 (166) parcellation.

Overall, Language was best modelled by CC defined with Shen (184) parcellation. Similarly, when FC was used to model Language it was also best defined with Shen (184) parcellation. However, when FS was used to model Language it was best defined with AAL3 (166) parcellation.

Overall, Encoding was best modelled with FC defined with Shen (278) parcellation. When SC was used to model Encoding, best models were achieved with AAL3 (166) and Shen (93). When CC was used to model Encoding, according to AIC differences, the preferred model was defined with Shen (184).

Finally, across connectivity modalities and parcellations, Sequence Processing was best modelled with Brainnetome (246). When either SC or CC was used, AIC values were in favour of models obtained with AAL3 (166).


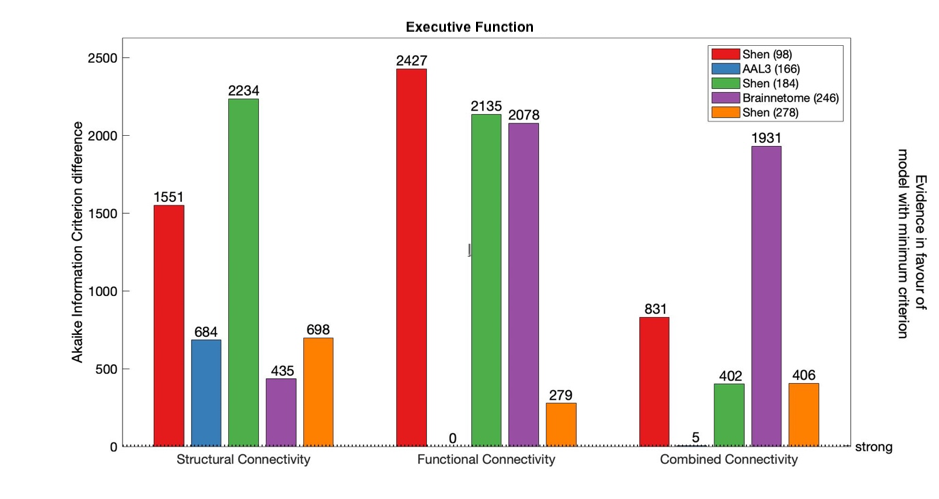


Figure 4 AIC difference for SC, FC and CC models of Executive Function, constructed with each parcellation scheme. Dotted line marks strong evidence in favour of FC model defined with AAL3 (166) (minimum AIC across all modalities and parcellations).


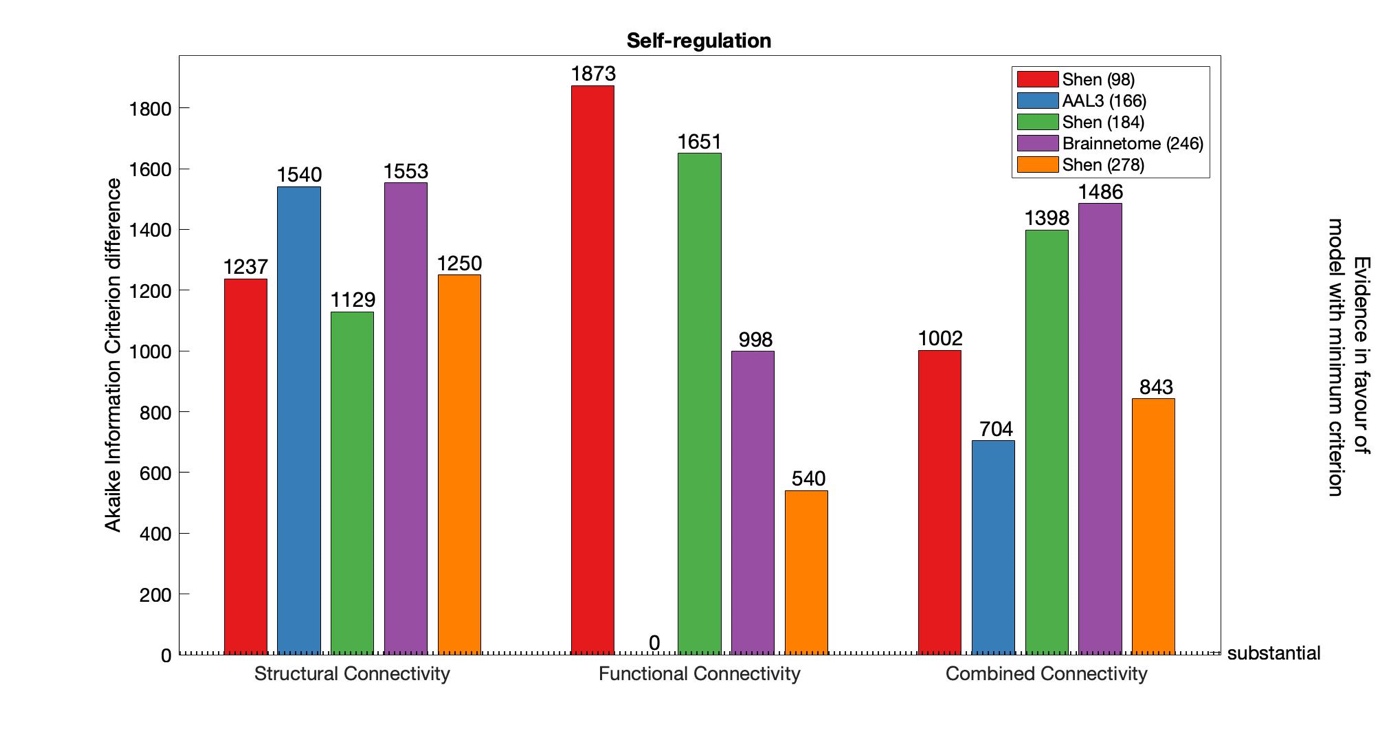


Figure 5 AIC difference for SC, FC and CC models of Self-regulation, constructed with each parcellation scheme. Dotted line marks substantial evidence in favour of FC model defined with AAL3 (166) (minimum AIC across all modalities and parcellations).
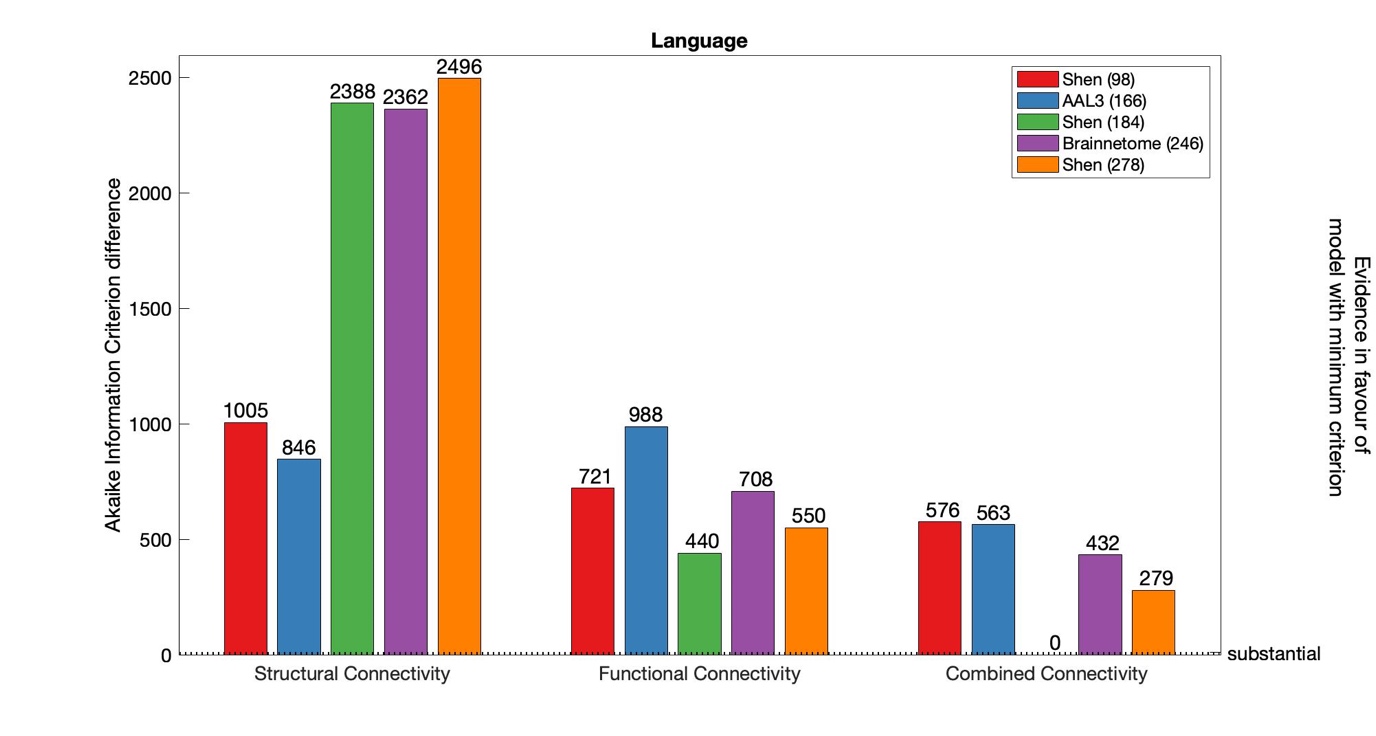


Figure 6 AIC difference for SC, FC and CC models of Language, constructed with each parcellation scheme. Dotted line marks substantial evidence in favour of CC model defined with Shen (278) (minimum AIC across all modalities and parcellations).


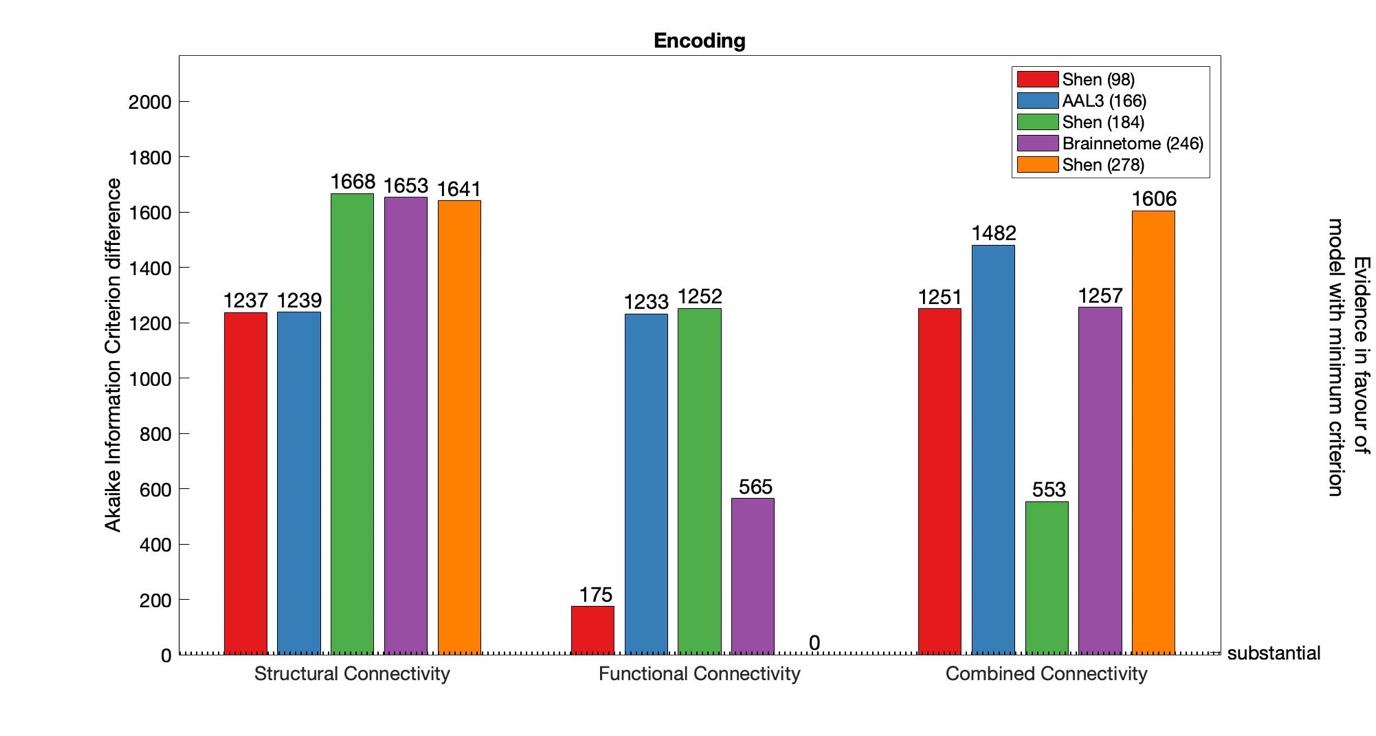


Figure 7 AIC difference for SC, FC and CC models of Encoding, constructed with each parcellation scheme. Dotted line marks substantial evidence in favour of FC model defined with Shen (278) (minimum AIC across all modalities and parcellations).


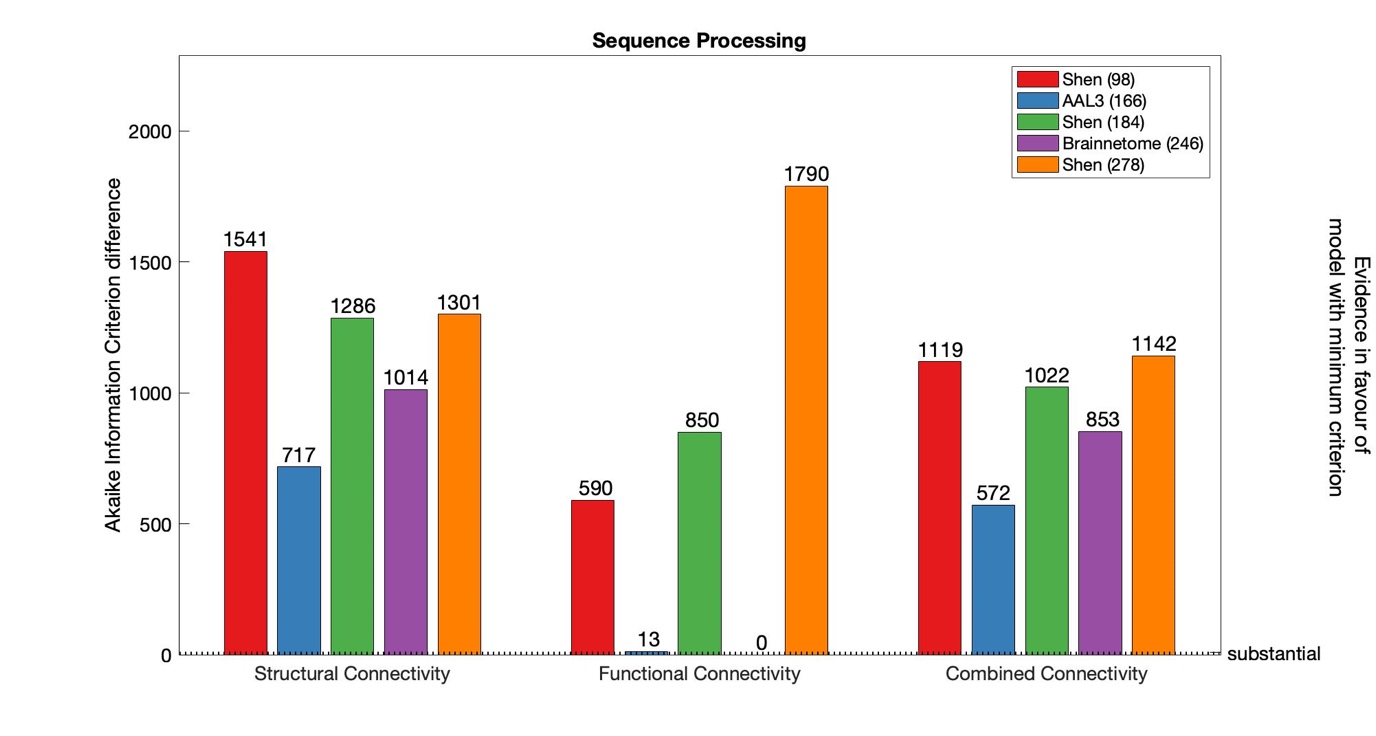


Figure 8 AIC difference for SC, FC and CC models of Encoding, constructed with each parcellation scheme. Dotted line marks substantial evidence in favour of FC model defined with Brainnetome (246) (minimum AIC across all modalities and parcellations).

# Graph theory

## Predictive modelling of demographics

#### AIC-based model comparison

Figures 9-11 demonstrate AIC for models of demographics composed with global graph theory measures of SC, FC and CC when defined with the 5 parcellation schemes.

AIC values demonstrated that across all connectivity modalities and parcellations, age was best modelled with FC defined Brainnetome (246) parcellation (Figure 19). For SC, there was weak evidence in favour of Shen (93 and 184) and Brainnetome (246) relative to AAL3 (166). For FC, there was weak evidence in favour of Brainnetome (246) parcellation to AAL3 (166) and Shen (278) and strong-to-substantial evidence in favour of Brainnetome (246) to Shen (93 and 184). For CC, there was strong-to-substantial evidence in favour of Brainnetome (246) parcellation to all alternatives.

Next, across connectivity modalities and parcellations, education had lowest AIC value when modelled with SC defined with Brainnetome (246) or with FC defined with Shen (93 and 184) parcellation. Within SC, there was strong evidence in favour of Brainnetome (246) relative to alternative parcellations. For FC, there was no notable difference between AIC values of models defined with Shen (184) and Shen (93). For CC, there was weak-to-substantial evidence in favour of Brainnetome (246) relative to alternative parcellations.

Finally, there was substantial evidence in favour of SC and CC models of sex relative FC models. For SC and CC, lowest AIC values were estimated for SC models defined with either Shen (184) followed by Shen (278). These two models were substantially favoured to any other alternative models.


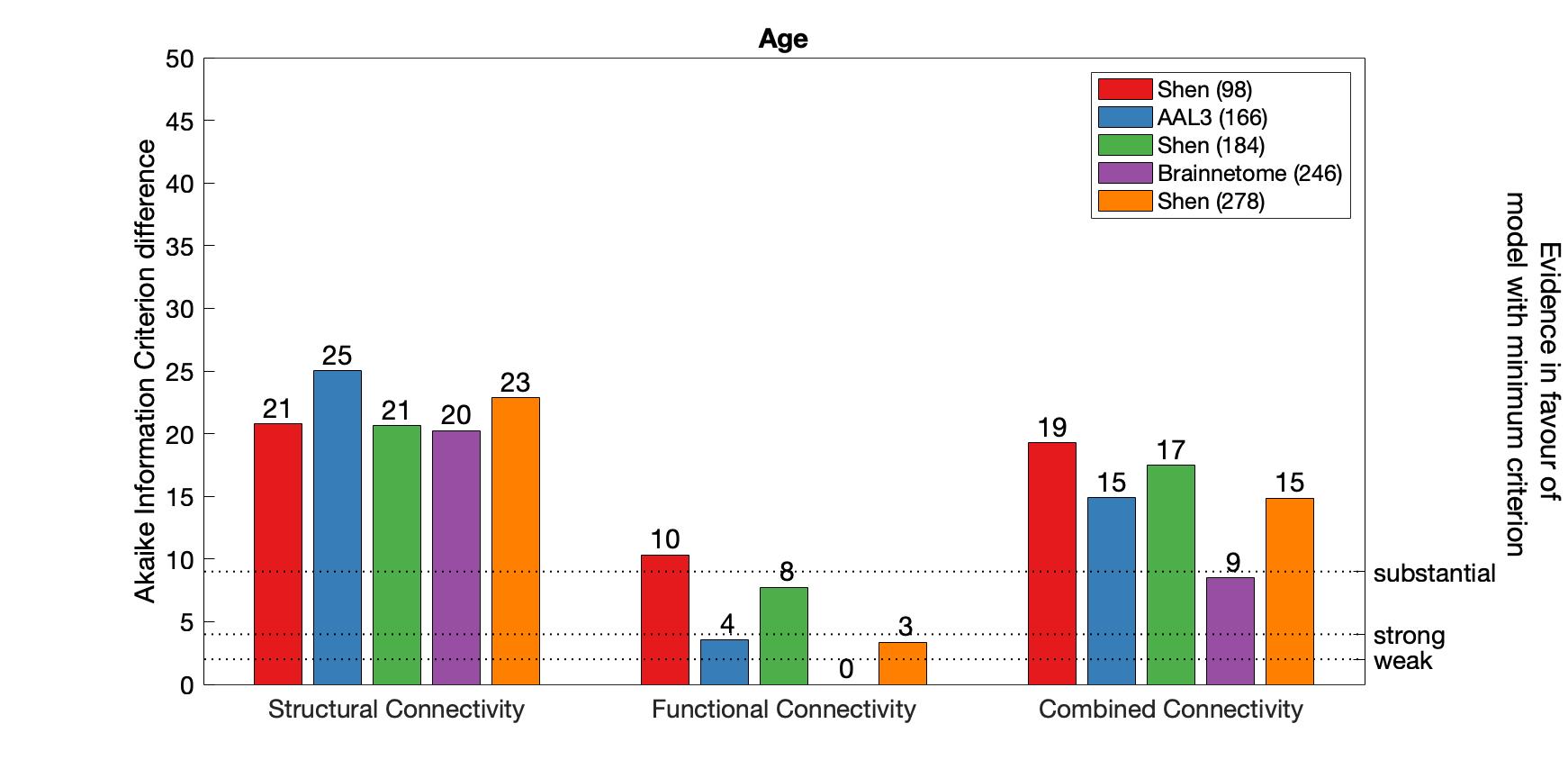


Figure 9 AIC difference for global graph theory measure models of Age, constructed with each parcellation scheme. Dotted line marks weak, strong and substantial evidence in favour of FC model defined with Brainnetome (246) (minimum AIC across all modalities and parcellations).


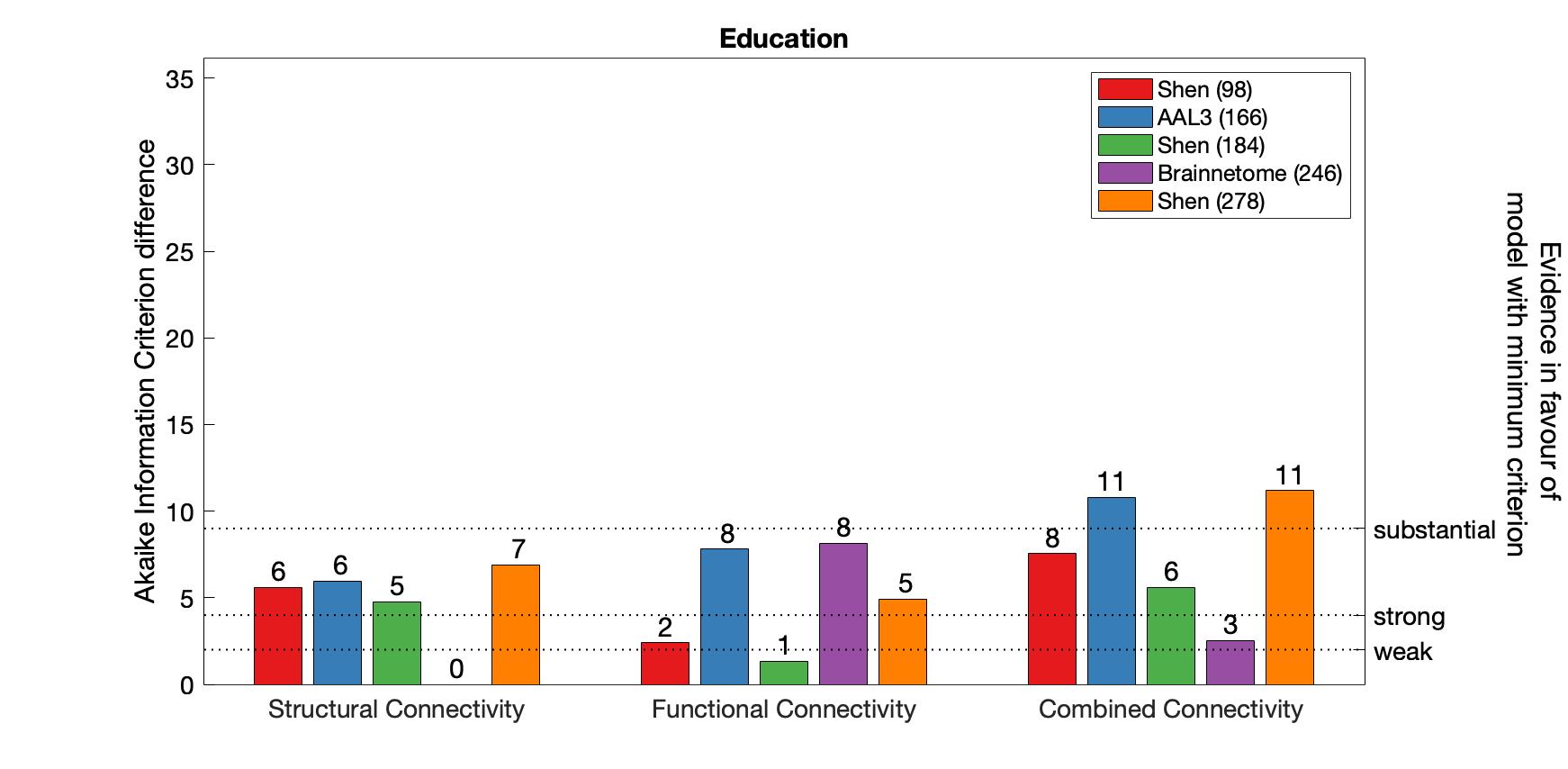


Figure 10 AIC difference for global graph theory measure models of Education, constructed with each parcellation scheme. Dotted line marks weak, strong and substantial evidence in favour of SC model defined with Brainnetome (246) (minimum AIC across all modalities and parcellations).


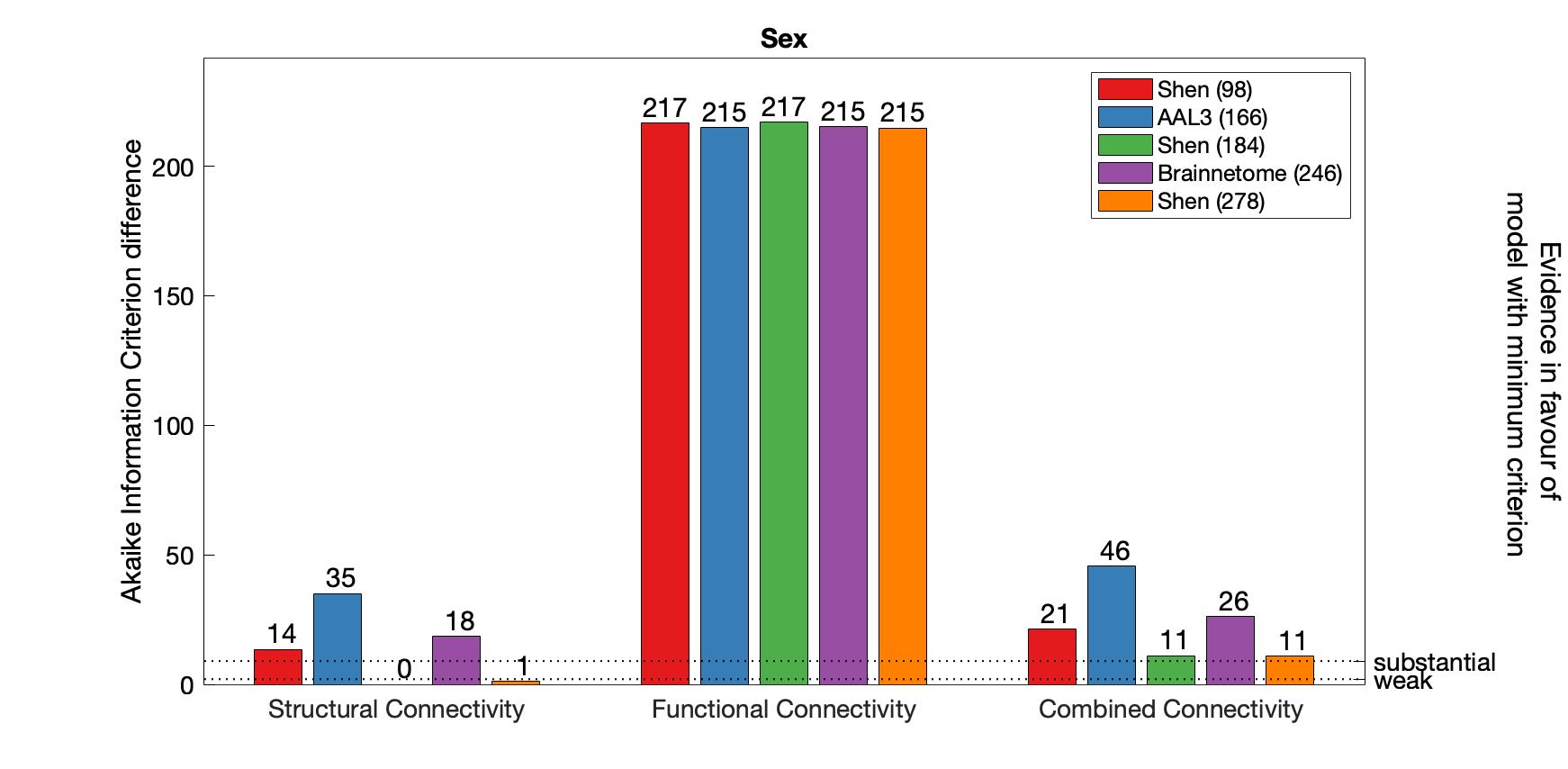


Figure 11 AIC difference for global graph theory measure models of Sex, constructed with each parcellation scheme. Dotted line marks weak, and substantial evidence in favour of SC model defined with Shen (184) (minimum AIC across all modalities and parcellations).

## Predictive modelling of cognition

#### AIC-based model comparison

Figures 12-16 demonstrate AIC for models of cognition composed with global graph theory measures of SC, FC and CC when defined with the 5 parcellation schemes.

Overall, AIC favoured models of Executive Function composed with SC defined with Shen (184) parcellation. In addition, there was no notable difference between SC, FC and CC models of Executive Function defined with Shen (184) parcellation. For SC, FC and CC, there was strong-to-substantial evidence in favour of Shen (184) relative to alternative parcellations.

Self-regulation was best modelled by SC when parcellated with Shen (278). For SC, there was no notable difference between AIC values of Shen (278) and Shen (93). However, there was weak evidence in favour of Shen (278) relative to Shen (184) and Brainnetome (246), and strong evidence relative to AAL3 (166). For FC, Self-regulation was best modelled with AAL3 (166) but the evidence in favour of this model relative to Shen (93 and 278) was not notable, and it was weak relative to Shen (184) and Brainnetome (246). In For CC, there was no notable difference between AIC values of Shen (93) and Shen (278). However, there was strong evidence in favour of Shen (278) relative to AAL3 (166) and Shen (184), and substantial evidence relative to Brainnetome (246).

Language was best modelled by SC defined with Shen (184) parcellation. For SC, there was no notable difference between AIC values of Shen (184) and Shen (278), but there was strong to substantial evidence in favour of Shen (184) relative to alternative parcellations. For FC, AIC difference weakly favoured AAL3 (166) parcellation relative to Shen (93, 184), and strongly relative to Brainnetome (246). For CC, there was no notable difference between AIC values of Shen (278) and Shen (184) and AAL3 (166) but there was strong-to-substantial evidence in favour of these models relative to Shen (93) and Brainnetome (246).

Overall, Encoding was best modelled with SC defined with Shen (93) parcellation. When FC was used to model Encoding, best models were achieved with either AAL3 (166) or Brainnetome (246). When CC was used to model Encoding, according to AIC differences, the preferred model was defined with AAL3 (93) and Shen (93).

Finally, Sequence Processing was best modelled with FC defined with either Shen (93) or Shen (184). When either SC or CC was used, AIC values were in favour of models obtained with Shen (184).


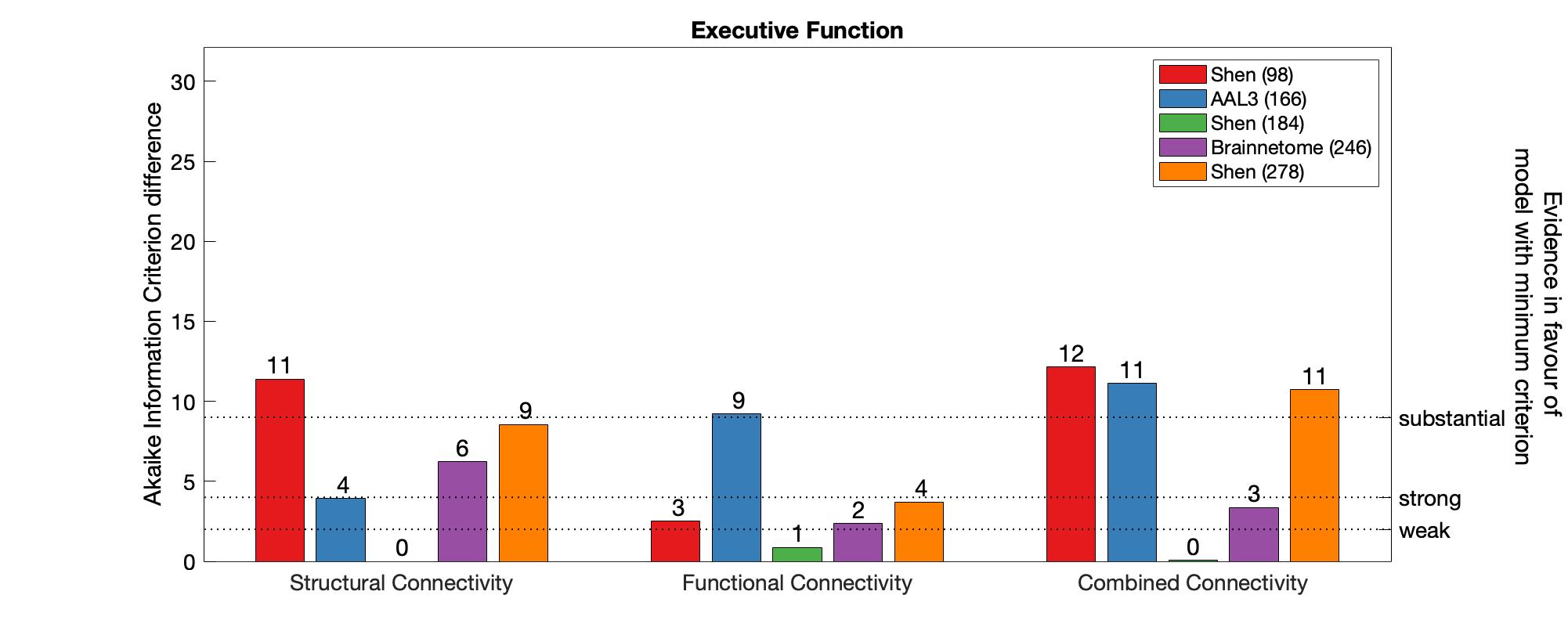


Figure 12 AIC difference for global graph theory measure models of Executive Function, constructed with each parcellation scheme. Dotted line marks weak, and substantial evidence in favour of SC model defined with Shen (184) (minimum AIC across all modalities and parcellations).


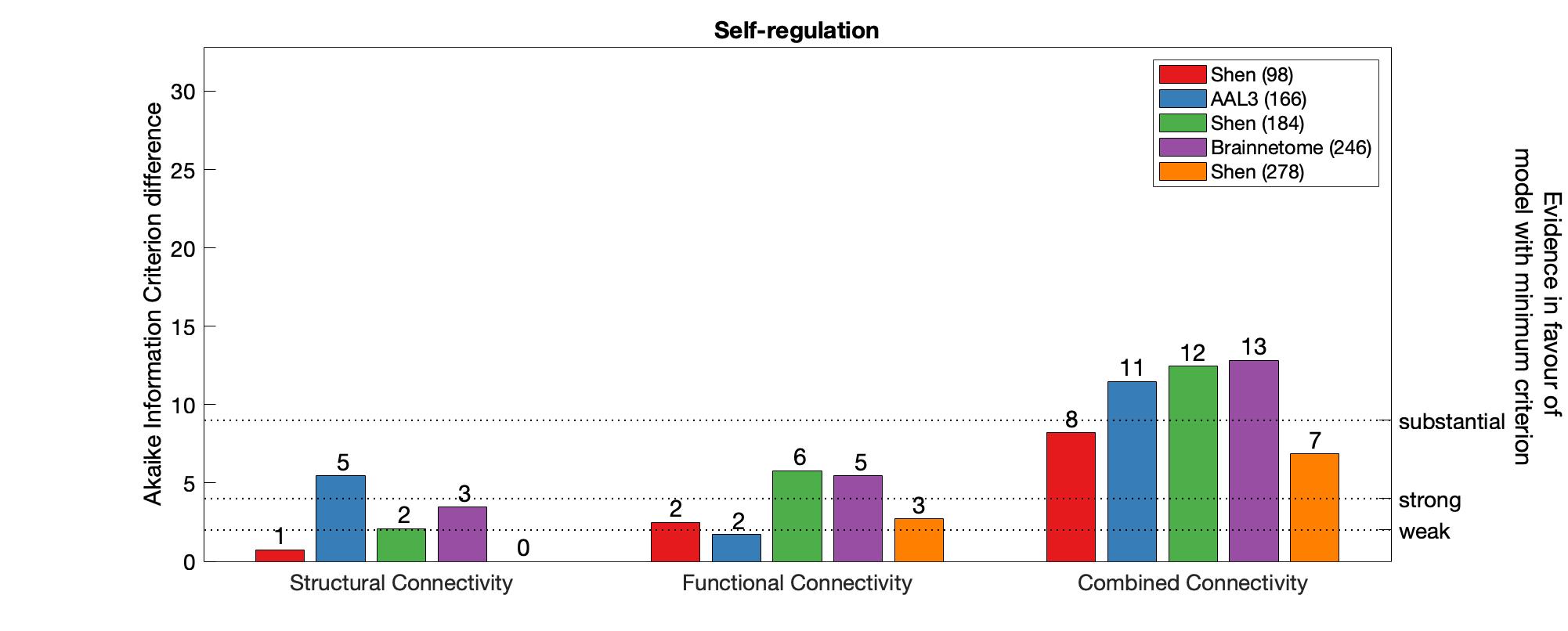


Figure 13 AIC difference for global graph theory measure models of Self-regulation, constructed with each parcellation scheme. Dotted line marks weak, and substantial evidence in favour of SC model defined with Shen (278) (minimum AIC across all modalities and parcellations).


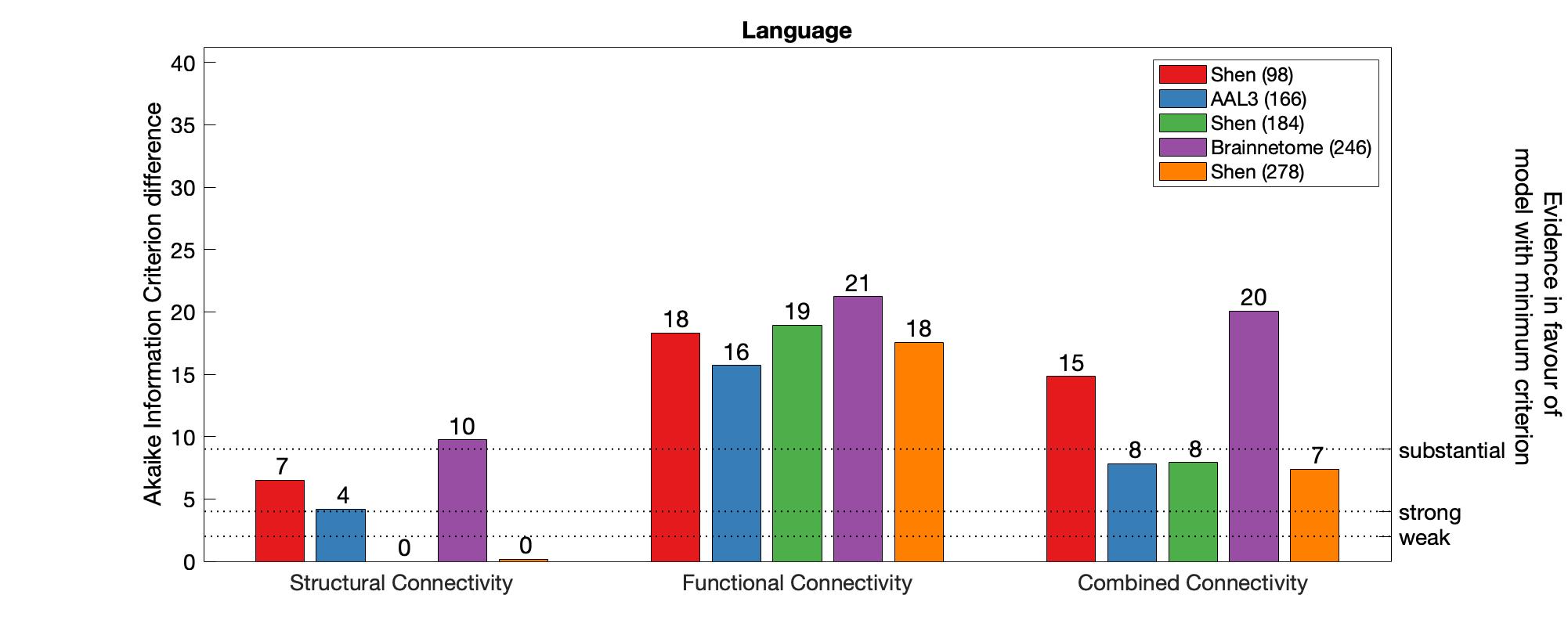


Figure 14 AIC difference for global graph theory measure models of Language, constructed with each parcellation scheme. Dotted line marks weak, and substantial evidence in favour of SC model defined with Shen (184) (minimum AIC across all modalities and parcellations).


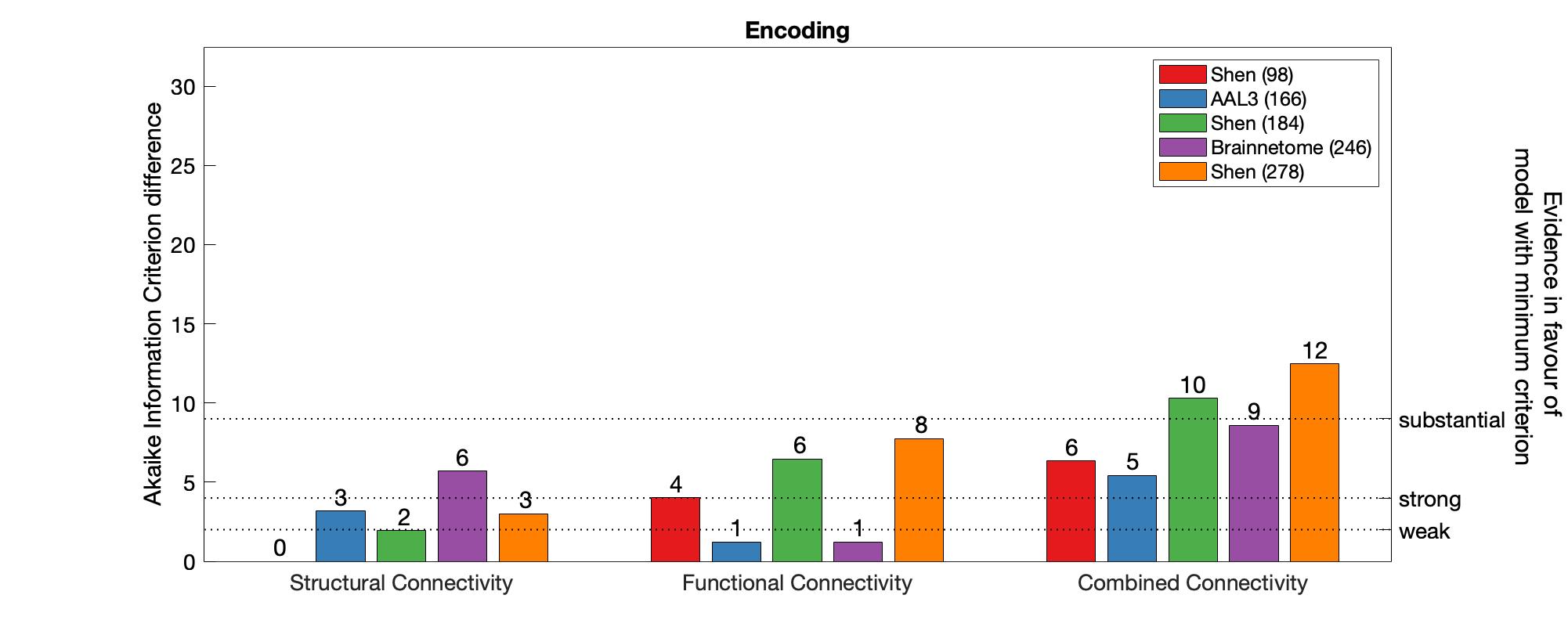


Figure 15 AIC difference for global graph theory measure models of Encoding, constructed with each parcellation scheme. Dotted line marks weak, and substantial evidence in favour of SC model defined with Shen (93) (minimum AIC across all modalities and parcellations).


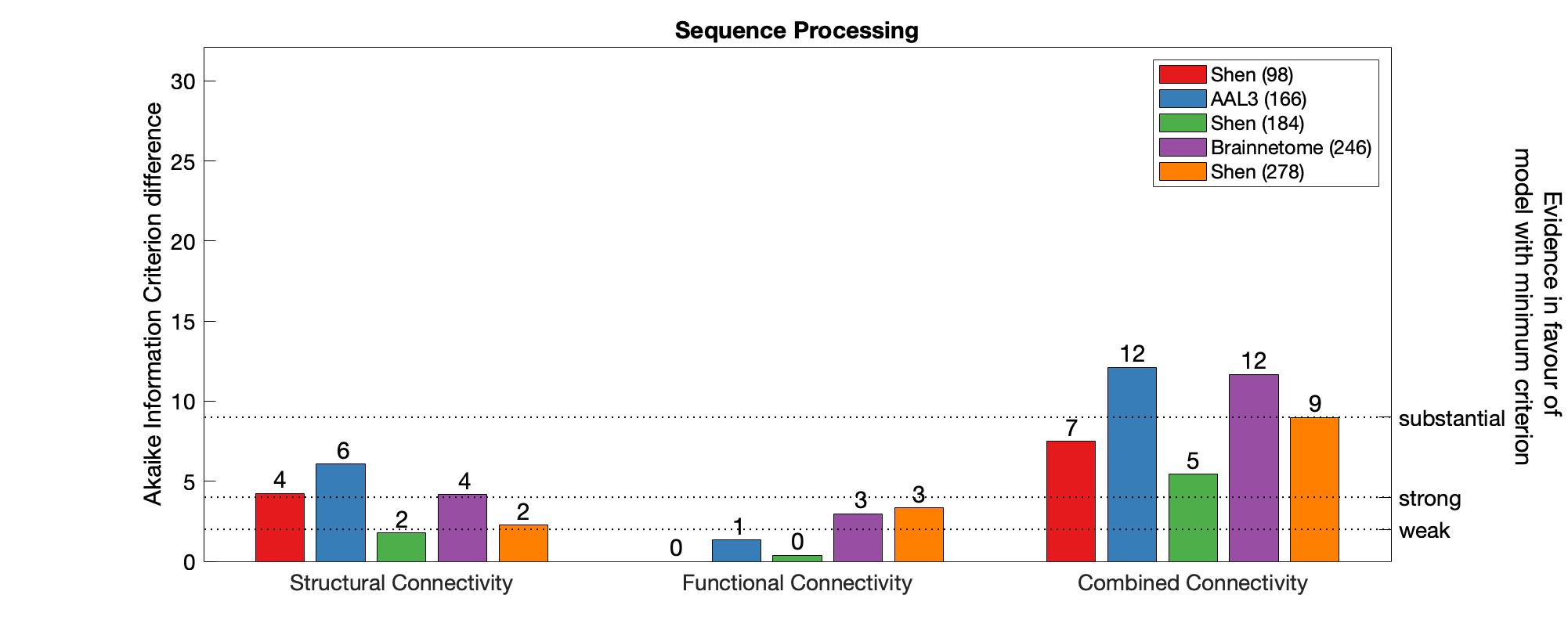


Figure 16 AIC difference for global graph theory measure models of Sequence Processing, constructed with each parcellation scheme. Dotted line marks weak, and substantial evidence in favour of SC model defined with Shen (93) (minimum AIC across all modalities and parcellations).

# Graph theory

### Comparison of global organisation across parcellations


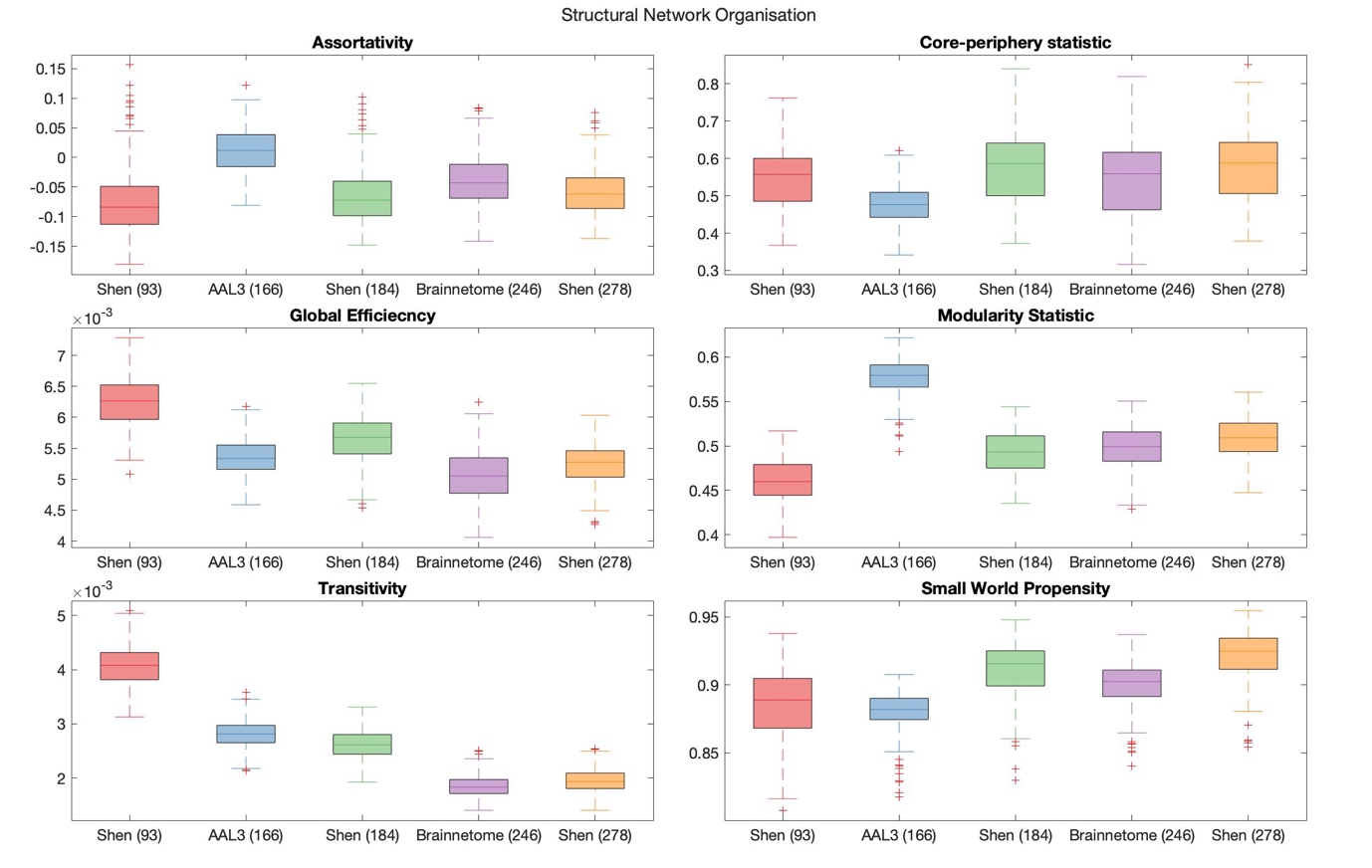


Figure 17 Graph theory measures of the global network organisation of SC.


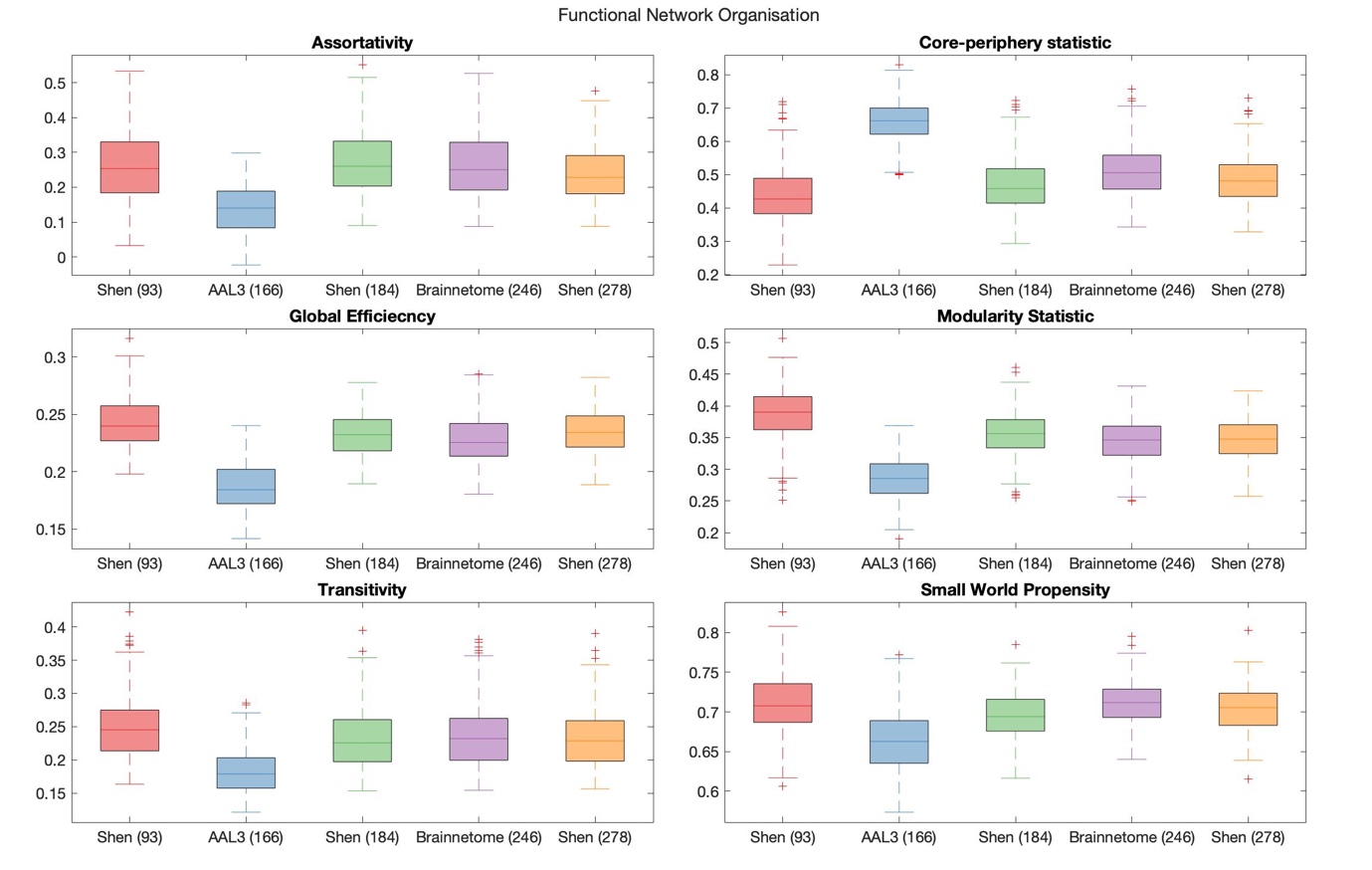


Figure 18 Graph theory measures of the global network organisation of FC.

Table 3 Pairwise comparison of t-statistics of structural connectivity organisation yielded by each network parcellation scheme. Parcellations presented in rows were used as the reference, therefore positive values reflect that the parcellation presented in a given row has higher global property (assortativity, core-periphery statistic, global efficiency, modularity, transitivity, small world propensity) than the parcellation presented in a given column, whereas negative values indicate that the parcellation presented in a given row has lower global property than the parcellation presented in a given column. All t-scores presented in bold have passed the FDR-adjusted critical p-value =<.0275.

Table 4 Pairwise comparison t-statistics of structural connectivity organisation yielded by each network parcellation scheme. Parcellations presented in rows were used as the reference, therefore positive values reflect that the parcellation presented in a given row has higher global property (assortativity, core-periphery statistic, global efficiency, modularity, transitivity, small world propensity) than the parcellation presented in a given column, whereas negative values indicate that the parcellation presented in a given row has lower global property than the parcellation presented in a given column. All t-scores presented in bold have passed the FDR-adjusted critical p-value =<.0275.

### Predictive modelling of demographics


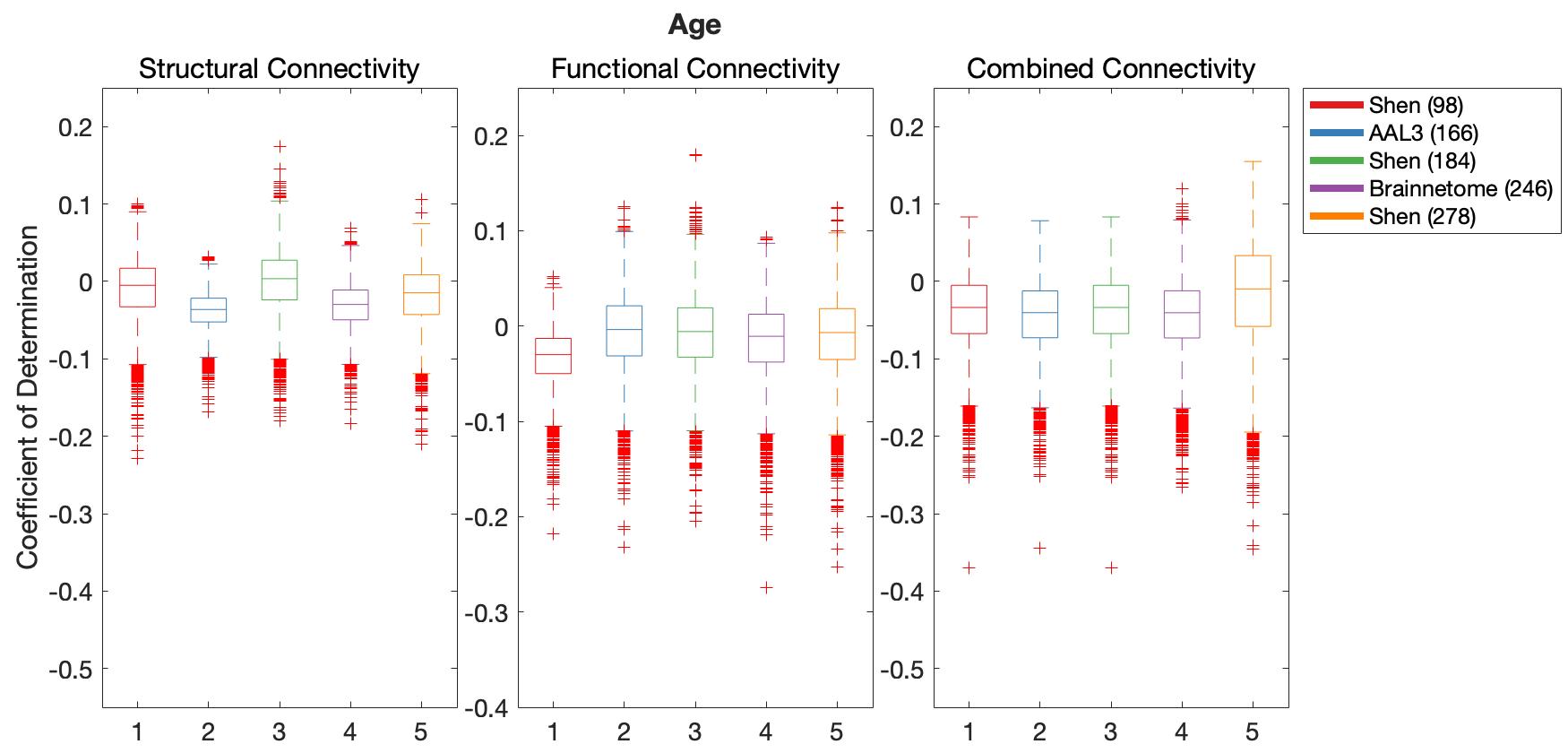


Figure 19 Results of BBC-CV of age models constructed with graph theory measures of SC, FC and CC, as measured by the coefficient of determination. The solid lines show the median scores, the boxes show the interquartile range (IQR), and ticks outside of whiskers indicate outlier scores across all bootstrap samples. Unfilled boxes illustrate below chance prediction.


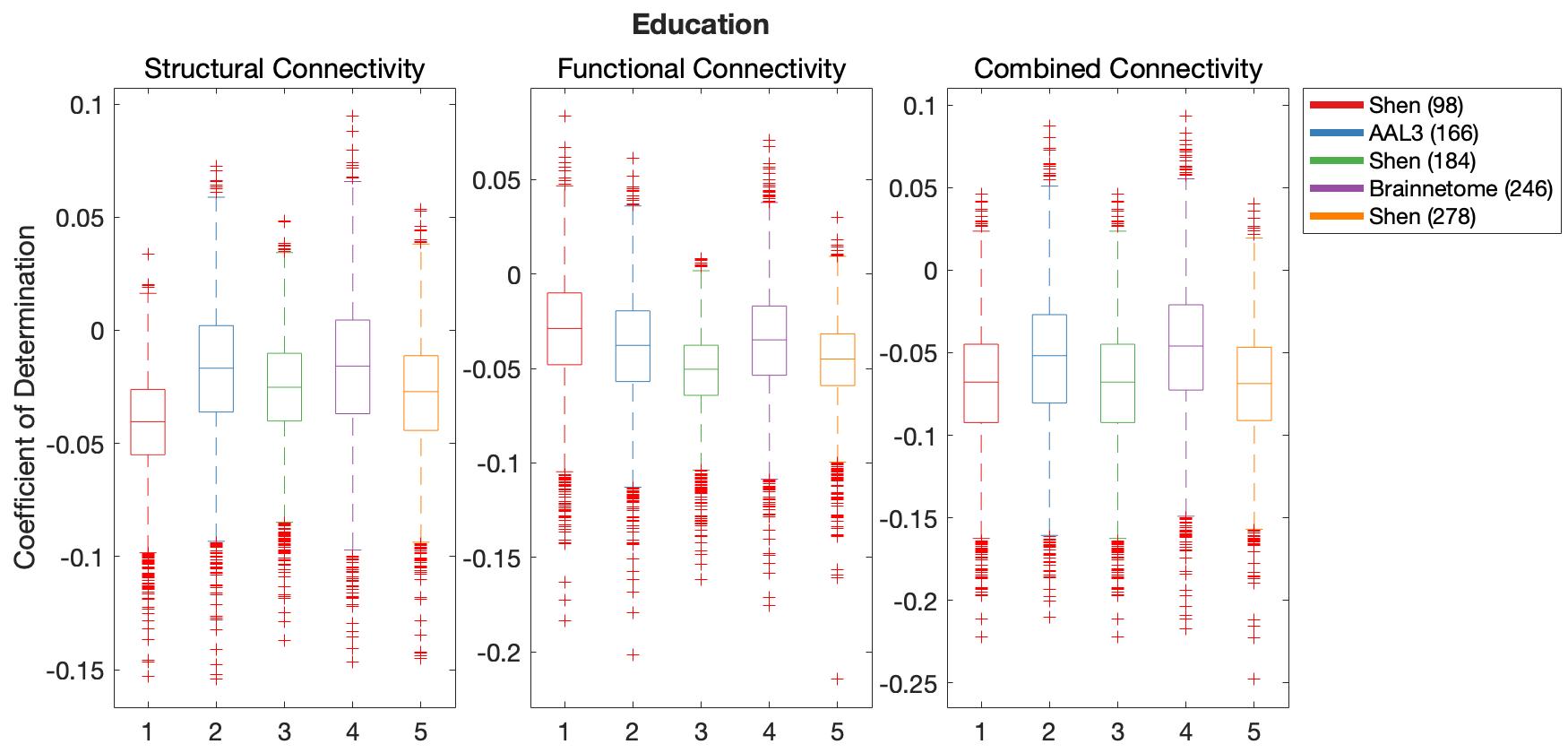


Figure 20 Results of BBC-CV of education models constructed with graph theory measures of SC, FC and CC, as measured by the coefficient of determination. The solid lines show the median scores, the boxes show the interquartile range (IQR), and ticks outside of whiskers indicate outlier scores across all bootstrap samples. Unfilled boxes illustrate below chance prediction.


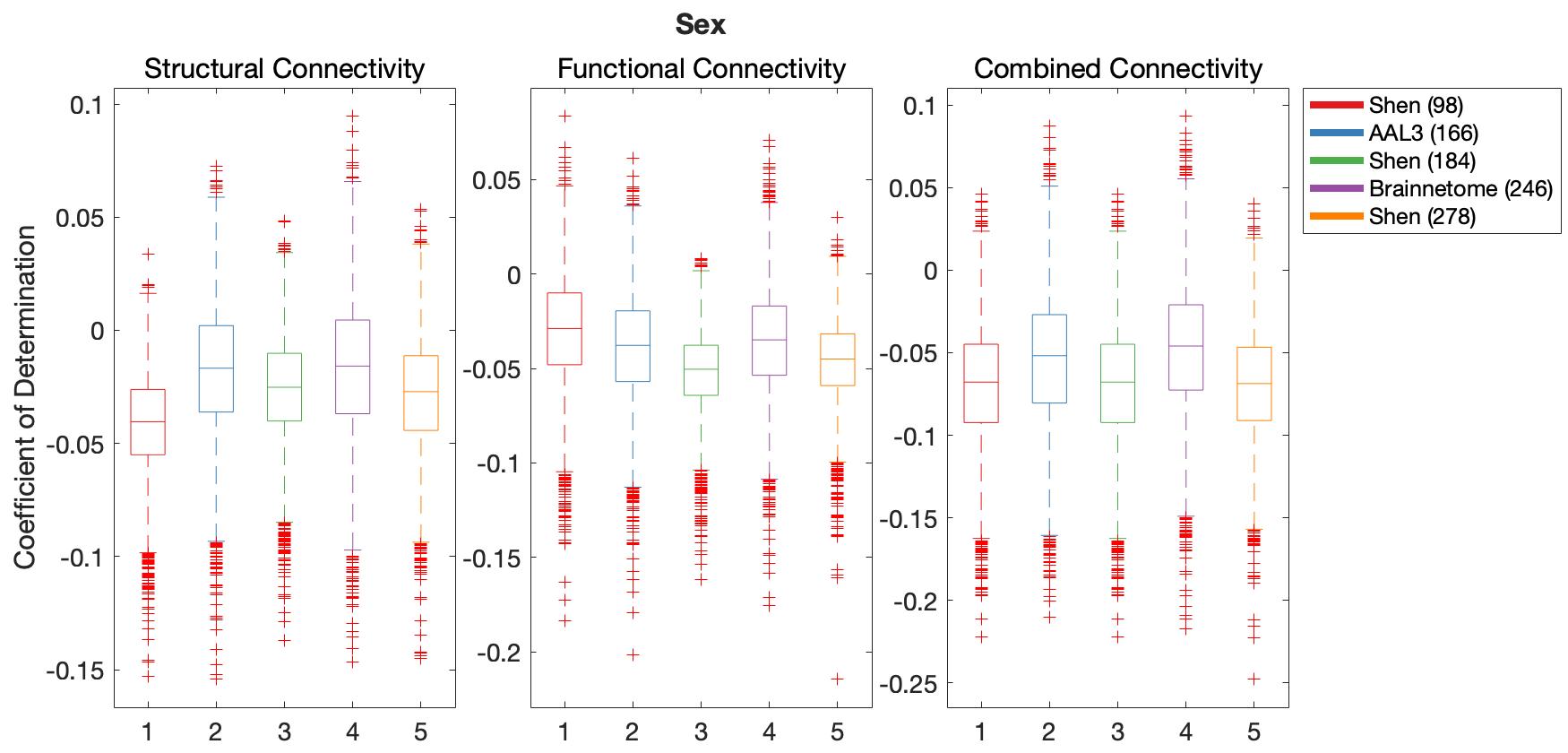


Figure 21 Results of BBC-CV of sex models constructed with graph theory measures of SC, FC and CC, as measured by the coefficient of determination. The solid lines show the median scores, the boxes show the interquartile range (IQR), and ticks outside of whiskers indicate outlier scores across all bootstrap samples. Unfilled boxes illustrate below chance prediction.

### Predictive modelling of cognition


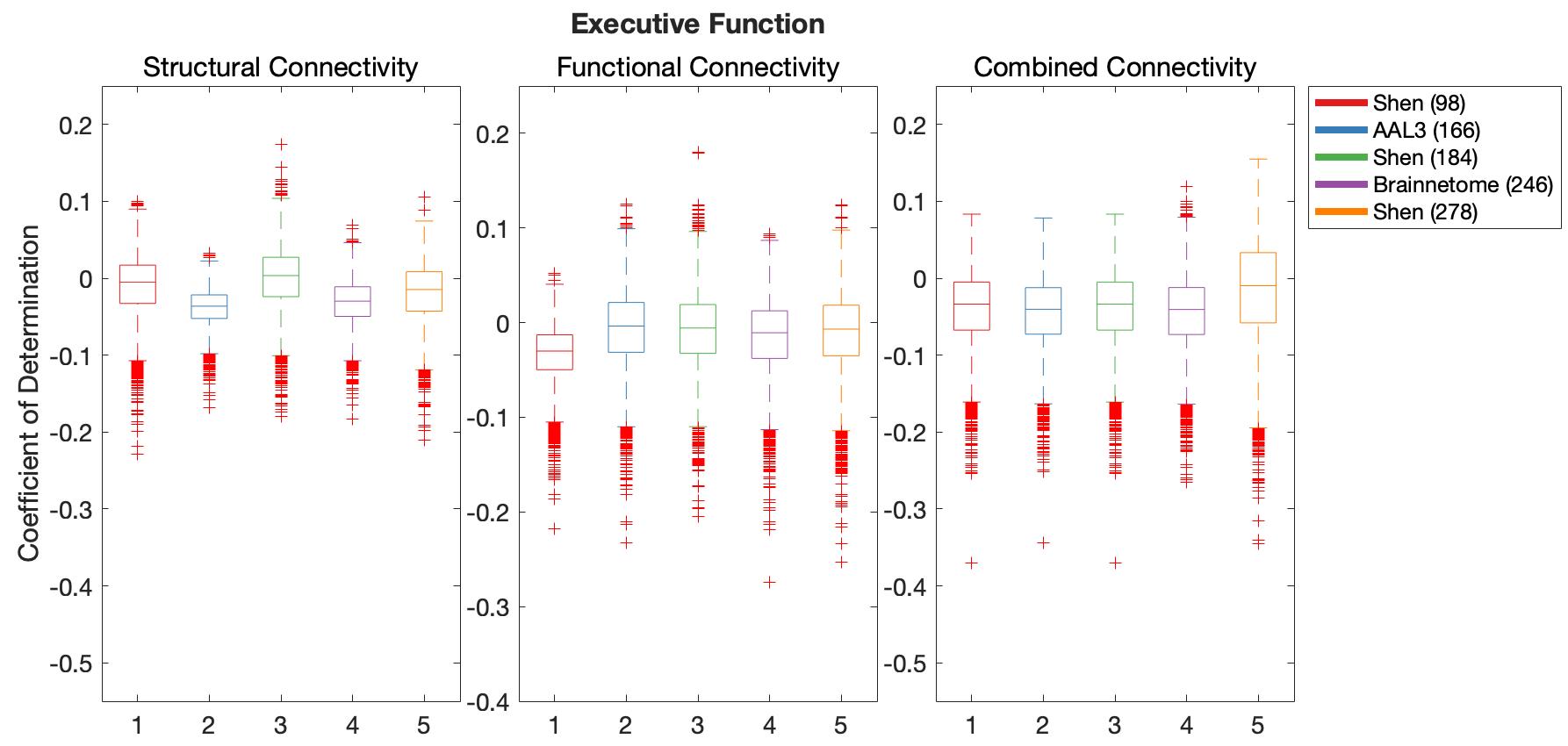


Figure 22 Results of BBC-CV of Executive Function models constructed with graph theory measures of SC, FC and CC, as measured by the coefficient of determination. The solid lines show the median scores, the boxes show the interquartile range (IQR), and ticks outside of whiskers indicate outlier scores across all bootstrap samples. Unfilled boxes illustrate below chance prediction.


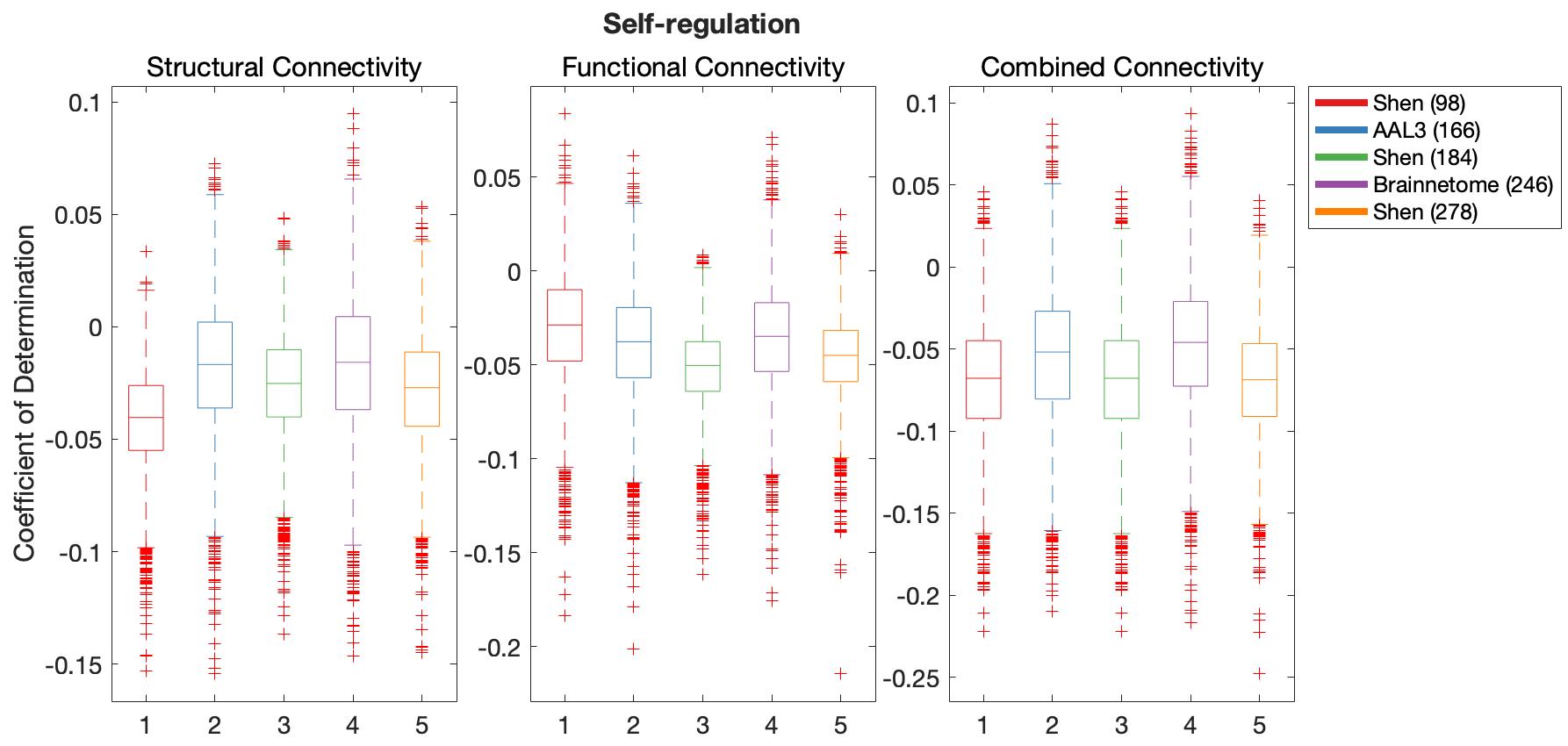


Figure 23 Results of BBC-CV of Self-regulation models constructed with graph theory measures of SC, FC and CC, as measured by the coefficient of determination. The solid lines show the median scores, the boxes show the interquartile range (IQR), and ticks outside of whiskers indicate outlier scores across all bootstrap samples. Unfilled boxes illustrate below chance prediction.


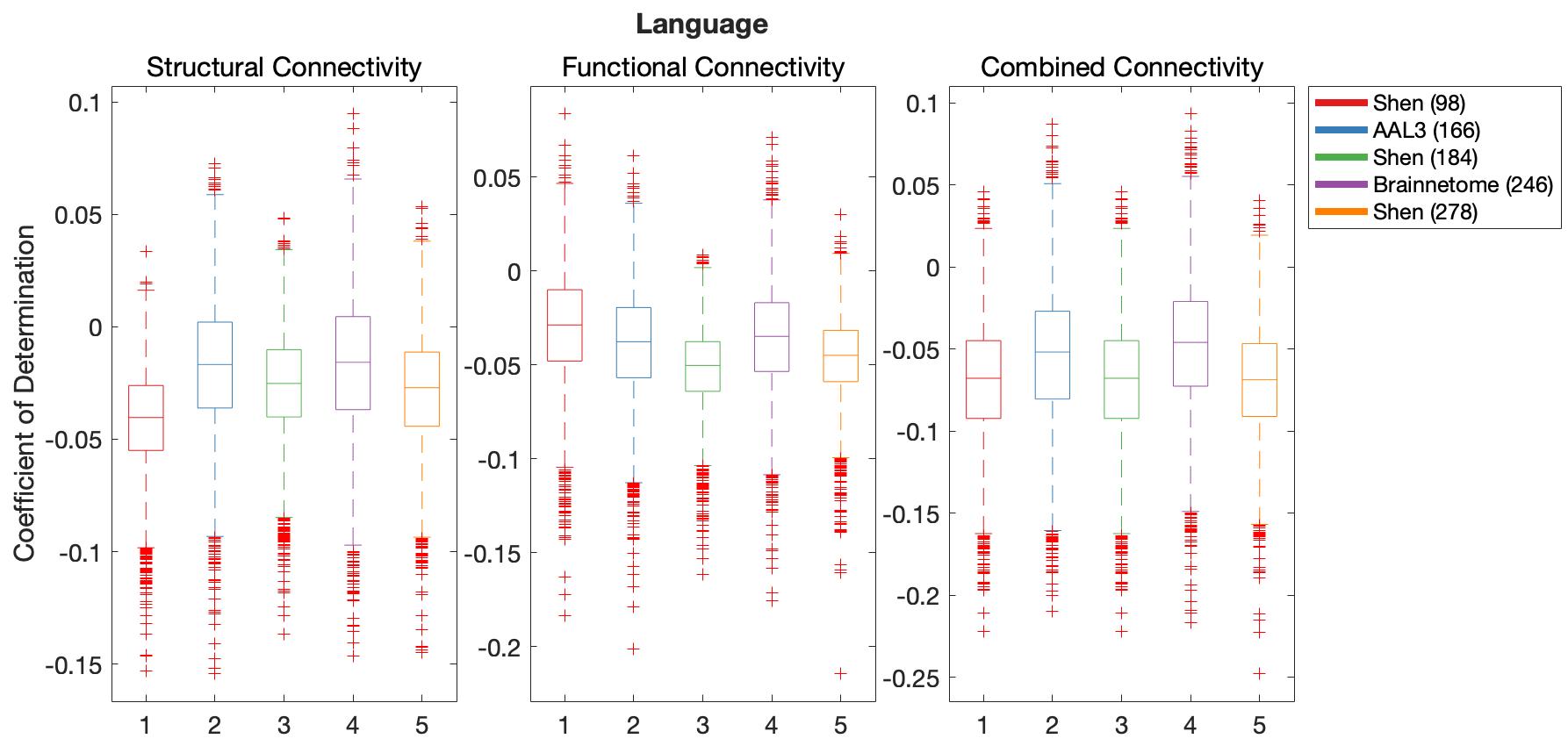


Figure 24 Results of BBC-CV of Language models constructed with graph theory measures of SC, FC and CC, as measured by the coefficient of determination. The solid lines show the median scores, the boxes show the interquartile range (IQR), and ticks outside of whiskers indicate outlier scores across all bootstrap samples. Unfilled boxes illustrate below chance prediction.


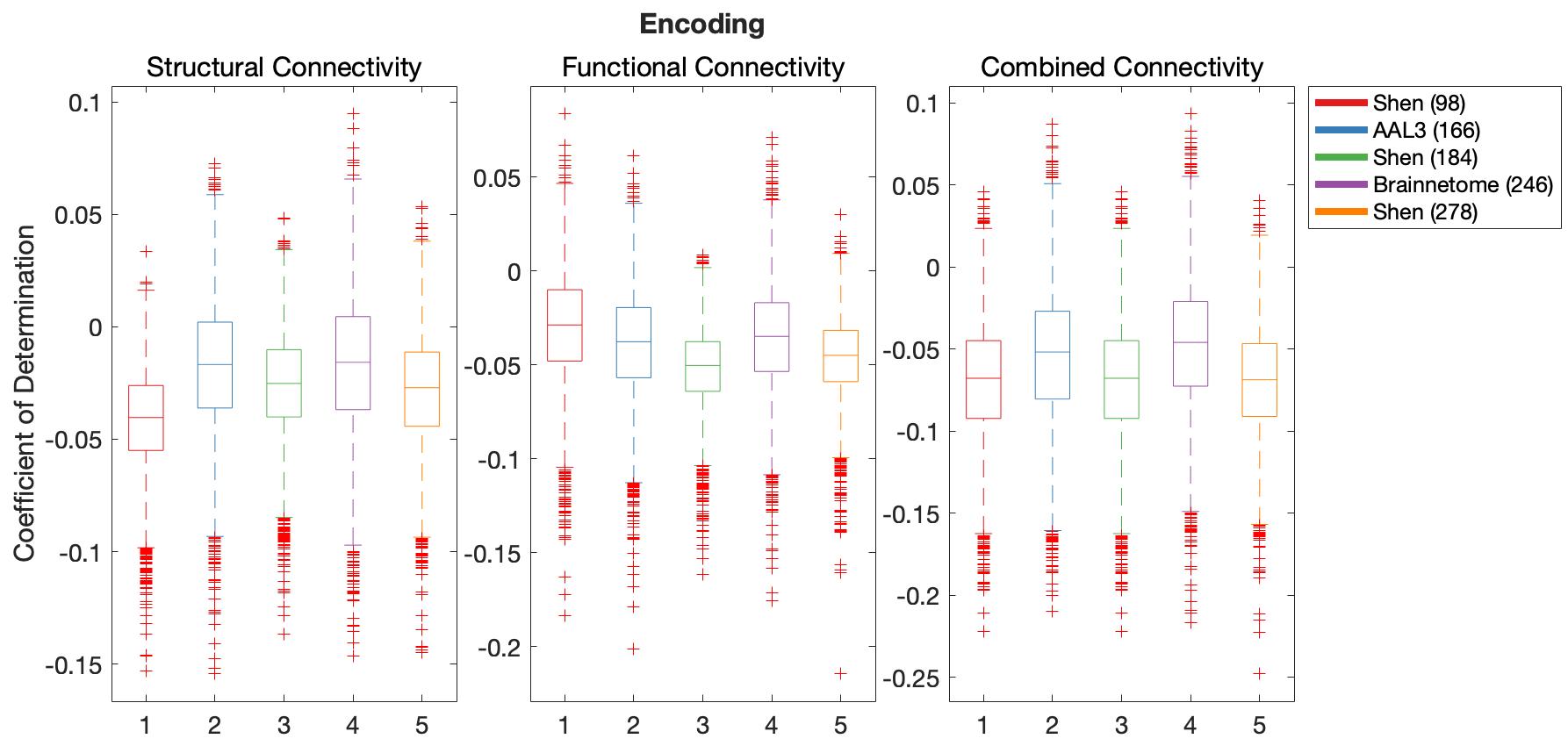


Figure 25 Results of BBC-CV of Encoding models constructed with graph theory measures of SC, FC and CC, as measured by the coefficient of determination. The solid lines show the median scores, the boxes show the interquartile range (IQR), and ticks outside of whiskers indicate outlier scores across all bootstrap samples. Unfilled boxes illustrate below chance prediction.


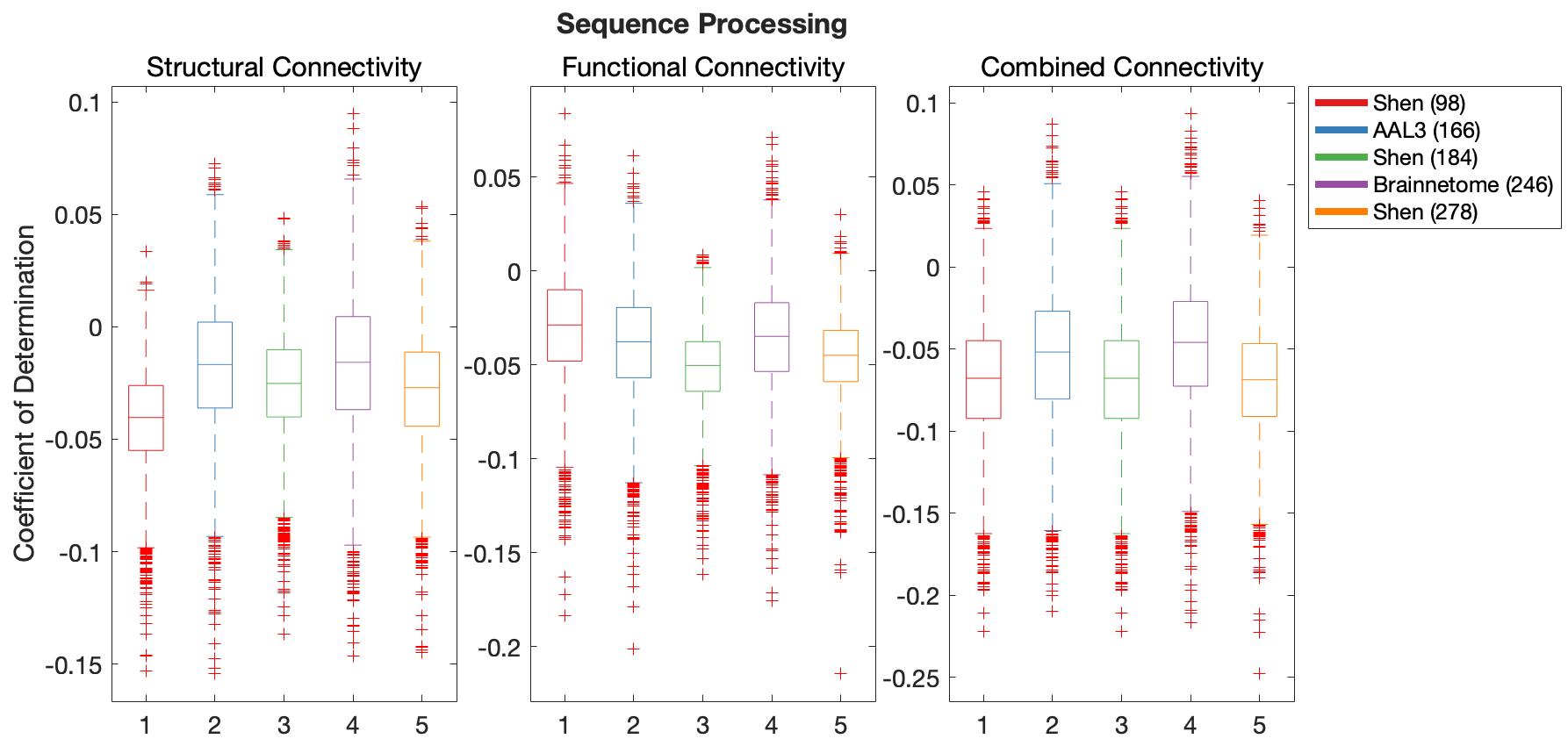


Figure 26 Results of BBC-CV of Sequence Processing models constructed with graph theory measures of SC, FC and CC, as measured by the coefficient of determination. The solid lines show the median scores, the boxes show the interquartile range (IQR), and ticks outside of whiskers indicate outlier scores across all bootstrap samples. Unfilled boxes illustrate below chance prediction.
